# Regional Variation in Mulberry Leaf Metabolites: A Combined Metabolomic and Environmental Analysis of Biosynthetic Drivers

**DOI:** 10.3390/metabo15110728

**Published:** 2025-11-06

**Authors:** Yao Zhou, Meiqi Li, Jinpeng Zhao, Lixia Yang, Fengxia Li, Jingtian Xu, Jingtian Chen, Yinyin Chen, Dongbei Xu, Dongju Feng, Wei Wu, Kai Hou

**Affiliations:** 1 College of Agronomy, Sichuan Agricultural University, Chengdu 611130, China; 201904859@stu.sicau.edu.cn (Y.Z.); 2024201063@stu.sicau.edu.cn (M.L.);; 2 Sichuan Provincial Rural Economic Information Center, Chengdu 610072, China; 3 Suining Meteorological Office, Suining 629094, China; 4 Neijiang Shizhongqu Agricultural and Rural Bureau, Neijiang 641000, China

**Keywords:** mulberry leaf, UHPLC-MS, environmental factors, metabolomics

## Abstract

**Background:** *Morus alba* L. (family Moraceae) is widely cultivated across the world and is well-known for its medicinal and nutritional value, especially its leaves. This study investigates the regional variation in mulberry leaf metabolites, focusing on alkaloids and flavonoids, and explores the influence of climatic and environmental factors on their biosynthesis using an integrated metabolomic and environmental analysis. Mulberry leaves, known for their medicinal and nutritional value, were collected from six regions across China, including Sichuan, Xinjiang, and Tibet. **Methods:** Untargeted metabolomics via UHPLC-MS was conducted. Differential metabolites were identified through multivariate analysis and annotated using the KEGG database. Redundancy analysis was used to link metabolite profiles with climatic data. **Results:** Mulberry leaves from six Chinese regions showed significant variation in total flavonoid content (TFC), total polyphenol content (TPC), and 1-Deoxynojirmycin (DNJ), with Tibet having the highest TFC and TPC, and Panzhihua the highest DNJ. Metabolomic analysis identified 3794 metabolites, revealing distinct regional clustering. A total of 79 differential metabolites were identified, which are enriched in pathways such as galactose metabolism and phenylalanine biosynthesis. Environmental factors, especially bio3, bio10, bio2, bio5, and bio20, strongly influenced metabolite profiles. **Conclusions:** The biosynthesis and accumulation of secondary metabolites in mulberry leaves are significantly influenced by region-specific environmental factors, particularly temperature, precipitation, and light. The identified differential metabolites are mainly enriched in galactose metabolism, arginine, and proline metabolism, and phenylalanine, tyrosine, and tryptophan biosynthesis. These pathways are closely associated with plant stress responses and the synthesis of secondary metabolites. The pronounced regional differences in metabolite profiles underscore the critical role of environmental factors in determining the chemical composition of mulberry leaves. This research provides valuable insights into the influence of climatic factors affecting the chemical composition of plants. It lays a theoretical foundation for the quality assessment and grading of mulberry leaves, providing scientific guidance for their targeted cultivation and utilization.

## 1. Introduction

*Morus alba* L. (family Moraceae) is a globally significant medicinal and edible plant with a millennia-long history of dual use in both traditional medicine and nutrition. It is cultivated globally, particularly across diverse regions in China, and demonstrates remarkable ecological adaptability. The leaf, the largest organ of the mulberry tree, accounts for approximately 60–70% of the plant’s total biomass. Revered as the “divine herb” in ancient Chinese texts such as the Shennong Ben Cao Jing (ca. 200 BCE), mulberry leaf was historically prescribed for treating *xiao-ke* (wasting thirst), a traditional Chinese medicine syndrome characterized by polyuria, polydipsia, and emaciation—symptoms now clinically associated with diabetes mellitus (DM). In the Ming Dynasty, Li Shizhen’s Compendium of Materia Medica (Bencao Gangmu, 1596 CE) documented that a daily mulberry leaf decoction, consumed as a tea substitute, was used to treat *xiao-ke*. The ancient Japanese monk Eisai, in his work Drinking Tea for Healthy (1211 CE), also recorded that mulberry leaves can help improve “thirsting disease” (which is equivalent to diabetes in modern medical terms). In Persian and Arab medicine, mulberry leaves were used to treat fever, cough, and skin diseases. Medieval European herbalists also recorded the medicinal value of mulberry leaves, believing that they could purify the blood and enhance immunity. Recent studies have confirmed the significant hypoglycemic potential of mulberry leaf, which is now widely incorporated into formulations for managing DM with demonstrable efficacy. Pharmacological studies have shown that mulberry leaf extracts and bioactive compounds—including flavonoids, polysaccharides, polyphenols and alkaloids (notably 1-Deoxynojirimycin, DNJ)—effectively lower blood glucose levels and mitigate DM [1,2,3,4]. The underlying mechanisms include modulation of glucose metabolism [5], protecting pancreatic β-cells [6], enhancement of insulin secretion [7], inhibition of enzymes such as α-glucosidase [8], and antioxidant effects. Collectively, these findings underscore the considerable therapeutic potential of mulberry leaf in both the prevention and treatment of DM.

The biosynthesis and accumulation of plant secondary metabolites are significantly regulated by environmental and climatic factors. These compounds play important roles in plant growth and development, environmental stress adaptation, and contribute to their medicinal properties. Recent studies have shown that ecological factors and soil conditions significantly affect the types and content of secondary metabolites in plants, including Astragalus (*Astragalus membranaceus*), mint (*Mentha pulegium* L.), *Salvia miltiorrhiza* (*Salvia miltiorrhiza* Bge.), *Erigeron breviscapus* (*Erigeron breviscapus* (Vant.) Hand.-Mazz.), and tea plants (*Camellia sinensis*) [9,10,11,12,13]. Geographical variables such as altitude, longitude, and latitude influence ecological factors such as temperature, humidity, and photoperiod, thereby indirectly modulating secondary metabolite biosynthesis [14,15]. For example, when plants are exposed to UV radiation or low-temperature environments, they generate reactive oxygen species (ROS), which can damage membrane structures, harm plant tissues, and cause metabolic disorders [16]. Active substances such as flavonoids, terpenoids, and polyphenols can promptly scavenge free radicals and enhance the plant’s antioxidant capacity under low-temperature conditions. UVR8 can sense these environmental signals and, through its interaction with HY5, activates the expression of a series of antioxidant enzymes and defense-related genes [17]. This process regulates the synthesis of hormones, polyphenols, flavonoids, and other substances within the plant, thereby enhancing its antioxidant and stress resistance capabilities. Other studies indicate that in *Draba oreades* Schrenk, the biosynthetic pathways of phenylpropanes and flavonoids are significantly upregulated with increasing growth altitude. The levels of flavonoids, including apigenin, luteolin, quercetin, and their glycoside derivatives, are indeed higher [18]. The accumulation of secondary metabolites in plants exhibits pronounced spatial and temporal variability across regions. Such variability contributes to inconsistency in the quality of plant-derived raw materials, posing a significant challenge to standardization and clinical reliability.

This study proposes the following hypothesis: Given the significant climatic heterogeneity of the sampling regions in Sichuan, Xinjiang, and Tibet, these differences will be systematically reflected in the content and types of secondary metabolites in mulberry leaves. Based on this, the study further proposes to use multivariate statistical methods to analyze the correlation between climate and metabolites, aiming to provide a scientific basis for the quality evaluation and geographical tracing of mulberry leaves. During the research, mulberry leaf samples were collected from six regions across three provinces in China—Sichuan, Xinjiang, and Tibet—and corresponding climatic data from their growth areas were obtained simultaneously. Metabolic profiling was conducted using UHPLC-MS to identify differential metabolites under diverse environmental conditions. Multivariate statistical analyses—including hierarchical cluster analysis (HCA), principal component analysis (PCA), correlation analysis, and redundancy analysis (RDA)—were used to explore associations between climatic variables and metabolite profiles. These findings provide valuable insights into the quantitative and qualitative influence of climatic factors on plant chemistry, and provide a theoretical and practical basis for the quality assessment and grading of mulberry leaves.

## 2. Materials and Methods

### 2.1. Plant Materials and Chemicals

#### 2.1.1. Collection and Processing of Plant Materials

**Figure 1:**
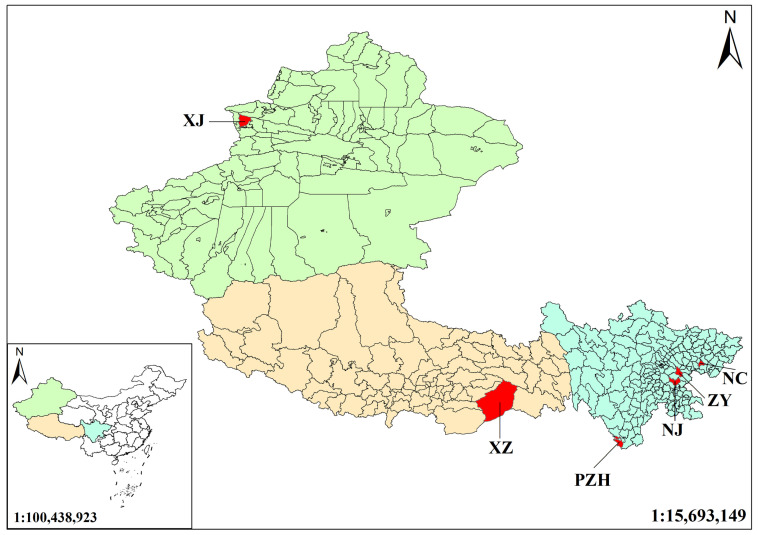
Geographic distribution of mulberry leaf sampling sites across China. Sampling locations are indicated in red. Abbreviations: NC–Gaoping District, Nanchong, Sichuan (30.7456° N, 106.1319° E); NJ–Zizhong County, Neijiang, Sichuan (29.7678° N, 104.8536° E); PZH–Renhe District, Panzhihua, Sichuan (26.495° N, 101.7503° E); ZY–Lezhi County, Ziyang, Sichuan (30.2808° N, 105.03° E); XJ–Huocheng County, Ili Kazak Autonomous Prefecture, Xinjiang (44.0489° N, 80.8458° E); XZ–Medog County, Nyingchi, Tibet (29.3128° N, 95.3175° E).

Tibet lies near the center of origin of Morus plants [19], whereas Xinjiang represents a key node along their diffusion route, and Sichuan harbors high genetic and ecological diversity. The selection of the mulberry leaves from Tibet, Xinjiang and Sichuan thus reflects the origin, diffusion, and evolutionary processes of Morus species, providing material for phylogenetic and biogeographic studies. The mulberry leaf samples used in this study were collected from six regions, including Sichuan, Xinjiang, and Tibet (Figure 1). Samples were collected from the same location in each region, with six replicates collected from each location, and all sampling was completed in August. All collected medicinal materials were authenticated as authentic samples according to pharmacognostic standards. The harvested mulberry leaves were dried at 60 °C to a constant weight, ground into powder and sieved through a 60-mesh sieve. The powdered samples were sealed in airtight containers and stored under controlled conditions.

#### 2.1.2. Chemicals

Rutin (CAS: 153-18-4), Gallic acid (CAS: 149-91-7), and 1-Deoxynojirimycin (CAS: 19130-96-2) were purchased from Push Bio-Technology Co., Ltd. (Chengdu, China). Folin–Ciocalteu reagent (Lot. 3541113001) was obtained from Solarbio (Beijing, China). Glycine (Lot. C16517696) was sourced from Macklin (Shanghai, China). Potassium borate buffer (Lot. JR30519B) and 9-Fluorenylmethyl chloroformate (FMOC-Cl, Lot. JS255491) were acquired from Shanghai Yuanye Biotechnology Co., Ltd. (Shanghai, China). All other chemicals were purchased from Chengdu Chron Chemicals Co., Ltd. (Chengdu, China).

### 2.2. Determination of Total Flavonoid Content (TFC), Total Polyphenol Content (TPC) and 1-Deoxynojirimycin (DNJ) in Mulberry Leaves

TFC was quantified using the aluminum nitrate method, TPC using the Folin–Ciocalteu method, and DNJ using the HPLC. Each experiment involved the measurement of three replicate samples, with each sample being measured three times.

#### 2.2.1. Total Flavonoid Content (TFC) [20]

Extraction Methods: A total of 0.25 g of mulberry leaf powder was mixed with 6.25 mL of 70% ethanol (*v*/*v*) and subjected to ultrasonic extraction at 70 °C for 15 min. The mixture was centrifuged at 5000 rpm for 10 min, and the supernatant was collected. The residue was re-extracted twice using the same procedure. All supernatants were combined and diluted to a final volume of 20 mL with 70% ethanol to obtain the total flavonoid extract. Assay Methods: A rutin standard was prepared in 70% ethanol and diluted to a series of concentrations: 200, 160, 120, 80, 40, 20, and 10 μg/mL. Subsequently, 120 μL of 5% NaNO_2_ solution was added to 2 mL of either the standard or sample solution and left to stand for 10 min. Then, 120 μL of 10% Al(NO_3_)_3_ solution was added and incubated for 10 min, followed by the addition of 1.6 mL of 4% NaOH. The final volume was adjusted to 4 mL with 70% ethanol. After zeroing the spectrophotometer (Shimadzu UV-2600, Kyoto, Japan) with 70% ethanol as the blank, absorbance was measured at 510 nm. The flavonoid concentration (μg/mL) in the reaction solution was calculated from the standard curve constructed using rutin concentration (x-axis) versus absorbance (y-axis). TFC was calculated based on the following formula.

$$\mathrm{TFC}\;(\mathrm{mg}/g)=\frac{V\times C\times D\times 10^{-3}}{M}$$

V–volume of the test solution (mL); C–the concentration of flavonoid (μg/mL) in reaction solution obtained from the standard curve; D–dilution ratio of flavonoids extraction solution; M–sample mass (g).

#### 2.2.2. Total Polyphenol Content (TPC) [21]

Extraction Methods: Mulberry leaf powder (0.5 g) was extracted with 50 mL of 70% ethanol (pH = 4.0) using ultrasonic treatment for 30 min. The mixture was then incubated in a 60 °C water bath for 90 min. After cooling, the mixture was centrifuged at 5000 rpm for 30 min. The resulting supernatant was collected as the total polyphenol extract. Assay Methods: A total of 2.5 mg of gallic acid standard was dissolved in distilled water to prepare a series of standard solutions (0, 4, 6, 8, 10, 12, 14, and 16 μg/mL). 1 mL of either the standard solution or sample solution was transferred to a centrifuge tube, 1 mL of Folin–Ciocalteu reagent was added and the mixture was incubated in the dark for 10 min. Subsequently, 2 mL of 10% Na_2_CO_3_ solution was added, and the mixture was further incubated in the dark at 25 °C for 1 h. The absorbance was measured at 765 nm using a UV spectrophotometer (Shimadzu UV-2600), and the instrument was zeroed with the reacted standard solution of zero concentration. A standard curve was constructed by plotting gallic acid concentration (x-axis) against absorbance (y-axis), from which the polyphenol concentration (μg/mL) in each reaction solution was determined. TPC was calculated based on the following formula and a standard curve was plotted.

$$\mathrm{TPC}\;(\mathrm{mg}/g)=\frac{V\times C\times D\times 10^{-3}}{M}$$

V–volume of the test solution (mL); C–the concentration of polyphenols (μg/mL) in reaction solution obtained from the standard curve; D–dilution ratio of polyphenol extraction solution; M–sample mass (g).

#### 2.2.3. 1-Deoxynojirimycin (DNJ) [22]

Extraction Methods: An amount of 0.25 g of mulberry leaf powder was mixed with 12.5 mL of 0.05 mol/L HCl and subjected to ultrasonic extraction for 30 min. The mixture was centrifuged at 4000 rpm for 30 min; the resulting supernatant was collected. The residue was re-extracted twice with 0.05 mol/L HCl solution using the same procedure. The combined supernatants were filtered and diluted to a final volume of 50 mL with 0.05 mol/L HCl solution. Assay Methods: DNJ standard (5 mg) was dissolved in 0.05 mol/L HCl to prepare a series of standard solutions at concentrations of 500, 50, 5, 0.5, and 0.05 μg/mL. A volume of 100 µL of either the standard or sample solution was mixed with 100 µL of 0.4 mol/L potassium borate buffer (pH = 8.5) and 200 µL of 5 mmol/L FMOC-Cl (dissolved in 50% acetonitrile). The mixture was incubated at 25 °C for 20 min. Subsequently, 100 μL of 1 mol/L glycine solution was added to terminate the reaction. Finally, 1 mL of 0.1% acetic acid was added, and the mixture was vortexed thoroughly. The solution was filtered through a 0.22 μm disposable syringe filter, and the resulting filtrate was collected for analysis. The standard curves were plotted with concentration on the x-axis and peak area on the y-axis.

Chromatographic conditions: Eclipse Plus C18 column (4.6 × 250 mm, 5 μm); UV detection wavelength: 254 nm; mobile phase: V(acetonitrile):V(0.1% acetic acid) = 40:60; flow rate: 1 mL/min; isocratic elution for 50 min; column temperature: 30 °C; injection volume: 10 μL.

$$\mathrm{DNJ}(\mathrm{mg}/g)=\frac{C\times V\times 10^{-3}}{M}$$

V–volume of the test solution (mL); C–the concentration of DNJ (μg/mL) in reaction solution obtained from the standard curve; M–sample mass (g).

### 2.3. Untargeted Metabolomics Analysis

To ensure experimental quality, QC samples were prepared by pooling equal volumes of all experimental samples and analyzed periodically to monitor system stability and performance. Blank samples, containing no analytes, were also analyzed to identify and remove background ions, ensuring the accuracy and reliability of the metabolomics data. A total of 100 mg of mulberry leaf powder (fresh-dried) was placed into Eppendorf tubes and mixed with 500 μL of 80% methanol, followed by vortexing to ensure full suspension. The samples were incubated on ice for 5 min and then were centrifuged at 15,000× *g*, 4 °C for 20 min. An aliquot of the supernatant was diluted with LC-MS-grade water to a final concentration of 53% methanol. The diluted samples were transferred to fresh Eppendorf tubes and centrifuged again at 15,000× *g*, 4 °C for 20 min. Finally, the resulting supernatant was injected into the UHPLC-MS/MS system for analysis. Quality control (QC) samples were prepared by mixing equal volumes of each experimental sample. Blank samples were prepared using a 53% aqueous methanol instead of experimental samples and were processed identically.

UHPLC-MS/MS analyses were performed using a Vanquish UHPLC system (Thermo Fisher, Bremen, Germany) coupled with an Orbitrap Q Exactive^TM^ HF mass spectrometer (Thermo Fisher, Bremen, Germany). Samples were injected onto a Hypersil Gold column (100 × 2.1 mm, 1.9 μm) using a 12 min linear gradient at a flow rate of 0.2 mL/min. Eluent A consisted of 0.1% formic acid in water, and eluent B was methanol. The solvent gradient was set as follows: 2% B, 1.5 min; 2–85% B, 3 min; 85–100% B, 10 min; 100–2% B, 10.1 min; 2% B, 12 min. Q Exactive^TM^ HF mass spectrometer was operated in positive/negative polarity mode with spray voltage of 3.5 kV, capillary temperature of 320 °C, sheath gas flow rate of 35 psi and aux gas flow rate of 10 L/min, S-lens RF level of 60, Aux gas heater temperature of 350 °C.

### 2.4. Multivariate Statistical Analysis, Kyoto Encyclopedia of Genes and Genomes (KEGG) Annotations and Metabolic Pathway Analysis of Differential Metabolites

Ion peaks with more than 50% missing values within each group were excluded. Positive and negative ion modes were integrated, and pattern recognition was performed using SIMCA-P 14.1 software (Umetrics, Umeå, Sweden). Following Pareto scaling, multivariate statistical analysis was performed, including principal component analysis (PCA) and partial least squares discriminant analysis (PLS-DA). Variable Importance in Projection (VIP) scores and *p*-values were used to identify significantly different metabolites among mulberry leaves from the six sampling regions. Significant metabolites were screened based on the criteria of VIP > 2 and *p* < 0.05. A 200-iteration permutation test was conducted to assess the risk of model overfitting.

Metabolite annotation was performed using the KEGG Compound database (https://www.genome.jp/kegg/compound/ (accessed on 12 November 2024)), and the resulting annotations were mapped to the KEGG Pathway database (https://www.kegg.jp/kegg/pathway.html (accessed on 12 November 2024)). Significantly enriched pathways were identified through metabolite set enrichment analysis, and statistical significance was determined using the hypergeometric test (*p* < 0.05).

### 2.5. Data on Climatic Factors

Climatic and geographical data—including longitude, latitude, altitude, and 20 bioclimatic variables—were collected from six regions across Sichuan, Xinjiang, and Tibet during the period from 2023 to 2024 (Supplementary Table S1). All climatic data were obtained from the Sichuan Provincial Rural Economic Information Center.

### 2.6. Data Processing

All data are expressed as Mean ± SD. Statistical significance was evaluated using one-way ANOVA followed by Duncan’s multiple range test, with *p* < 0.05 considered statistically significant. Statistical analyses were conducted using SPSS version 28.0 (IBM Corp., Chicago, IL, USA). Redundancy analysis (RDA) was performed using Canoco 5.0 to evaluate the relationship between mulberry leaf metabolites and climate factors. The contributions of environmental variables to metabolite variation were quantified by RDA and visualized along the x- and y-axes in the ordination plots. Pearson correlation analysis method was performed to assess the relationship between metabolites and environmental factors across different mulberry-producing regions.

## 3. Results

### 3.1. Variation in TFC, TPC, and DNJ Contents in Mulberry Leaves from Different Regions

TFC, TPC, and DNJ contents were quantified to evaluate variations in the chemical composition of mulberry leaves across six cultivation regions (Table 1). The standard curves for TFC, TPC, and DNJ were constructed using rutin, gallic acid, and DNJ standards, respectively. The linear regression equations and correlation coefficients (R^2^) for the standard curves were as follows: TFC (Rutin): y = 0.0065x − 0.0028, R^2^ = 0.9998; TPC (Gallic acid): y = 0.0397x − 0.0031, R^2^ = 0.9995; DNJ: y = 2137.8x + 5698.1, R^2^ = 0.9996. These high R^2^ values indicate good linear relationships between the concentrations of the standards and their peak areas, validating the accuracy of the quantification methods.

The sample from XZ exhibited the highest TFC (71.94 ± 2.04 mg/g) and TPC (30.63 ± 0.41 mg/g), significantly exceeding the levels observed in other regions. However, it contained the lowest DNJ content (0.14 ± 0.01 mg/g). PZH showed the second-highest TFC (41.13 ± 0.40 mg/g) and TPC (15.90 ± 0.37 mg/g), and also exhibited the highest DNJ content (3.65 ± 0.03 mg/g). ZY exhibited moderate levels of TFC (30.50 ± 1.18 mg/g) and TPC (14.65 ± 2.67 mg/g), but a relatively low DNJ content (0.45 ± 0.07 mg/g). XJ and NJ contained intermediate TFC (19.86 ± 0.47 and 12.99 ± 0.55 mg/g) and TPC (10.24 ± 0.45 and 8.29 ± 0.01 mg/g, respectively), along with relatively low DNJ levels (0.80 ± 0.05 and 0.75 ± 0.11 mg/g), respectively. NC contained low levels of low TFC (14.46 ± 0.58 mg/g) and TPC (6.61 ± 0.54 mg/g), but a higher DNJ content (1.99 ± 0.17 mg/g), second only to that observed in PZH. These findings indicate substantial regional variation in the accumulation of flavonoids, polyphenols, and DNJ in mulberry leaves.

### 3.2. Metabolomics Reveals Distinct Geographical Clustering of Mulberry Leaves Through Multivariate Statistical Analysis

The total ion chromatogram (TIC) of the mulberry leaf samples showed a stable baseline with well-distributed peaks and high resolution, meeting the requirements for component separation (Supplementary Figure S1). The TIC curves of the three QC samples overlapped closely, demonstrating consistent retention times and peak intensities, which indicates the detection platform’s excellent signal stability when analyzing the same sample at different time points (Supplementary Figure S2).

**Figure 2:**
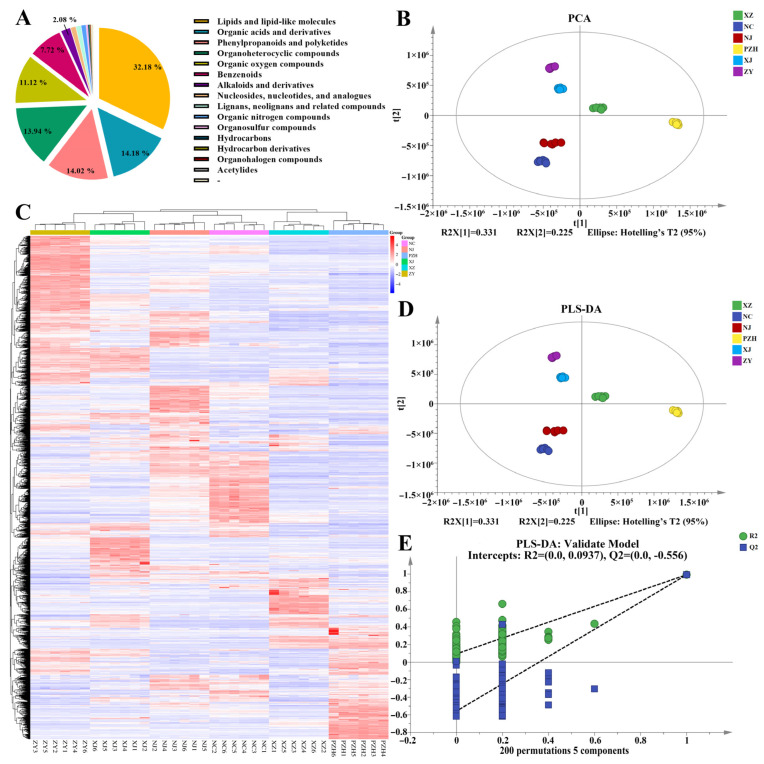
(**A**) Classification of the 3794 metabolites in mulberry leaves samples. Nucleosides, nucleotides, and analogues, as well as the proportions of the subsequent categories, are as follows: 1.13%, 1.08%, 1.03%, 0.40%, 0.32%, 0.16%, 0.11%, 0.03%, 0.50%; (**B**) Principal component analysis (PCA) score plot; (**C**) Hierarchical cluster analysis (HCA) of all metabolites across samples, with each sample represented by a column and each metabolite by a row. (**D**) Partial least squares discriminant analysis (PLS-DA) score plot; (**E**) Permutation test plot validating the PLS-DA model. The dotted lines represent the regression lines for R^2^Y or Q^2^. If the regression lines show an upward trend, it indicates that the permutation test has passed, and the model does not exhibit overfitting. Conversely, if the regression lines show a downward trend, it suggests that the model does exhibit overfitting.

Metabolomic analysis using UHPLC-MS/MS revealed distinct metabolite profiles in mulberry leaves collected from different geographical regions. A total of 3794 metabolites were identified in mulberry leaves, representing a diverse phytochemical profile. Lipids constituted the most abundant category, accounting for 32.18% of the total metabolites, followed by organic acids (14.18%), phenylpropanoids (14.02%), and organoheterocyclic compounds (13.94%). Organic oxygen compounds and benzenoids represented 11.12% and 7.72%, respectively, while alkaloids accounted for 2.08%. The remaining eight metabolite classes collectively represented less than 5% of the total composition (Figure 2A).

PCA clearly distinguished samples from different regions. The first two principal components (PC1 and PC2) explained 33.1% and 22.5% of the total variance, respectively, cumulatively accounting for 55.6% of the variance (Figure 2B). Notably, samples from each region formed distinct clusters, indicating pronounced geographic variation in metabolite composition. Hierarchical cluster analysis (HCA) further supported these findings, revealing that samples from PZH and XZ exhibited high metabolic similarity and clustered together, while samples from other regions—including XJ—formed separate groups (Figure 2C).

To enhance classification and interpretation, PLS-DA was employed (Figure 2D). The model performed robustly, with R^2^X = 0.903, R^2^Y = 0.991, and Q^2^ = 0.989, indicating high explanatory and predictive capability. Score plots showed clear separation among all six regions, including XZ, XJ, and PZH, confirming significant inter-regional metabolic differences. A permutation test with 200 iterations validated the model, yielding R^2^ and Q^2^ intercepts of 0.0937 and −0.556, respectively, demonstrating that the model was not overfitted (Figure 2E).

These results underscore the substantial influence of geographical origin on the metabolic profile of mulberry leaves, with distinct patterns observed for XZ, XJ, and PZH, among other regions.

### 3.3. Identification and Analysis of Differential Metabolites in Mulberry Leaves from Different Regions

**Figure 3:**
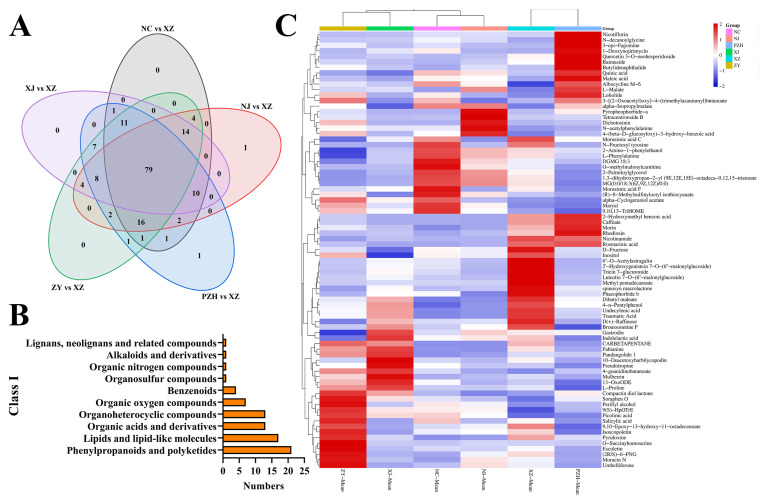
(**A**) Venn diagram of differential metabolites; (**B**) Differential metabolites classification diagram (Class I); (**C**) Heatmap results of 79 differential metabolites in mulberry leaves samples from six regions. Each row represents a metabolite, and each column represents the differential metabolites in mulberry leaves samples from different regions. Red indicates high content of metabolites, and blue indicates low content of metabolites.

The variable importance of projection (VIP) value reflects the contribution of each compound to the group separation in the PLS-DA model. A higher VIP value indicates a greater contribution to classification and a more marked variation in compound abundance across regions. To identify compounds with substantial variation among mulberry leaves from the six regions, metabolites with VIP > 2 and *p* < 0.05 were selected as differential. A total of 79 common differential metabolites were identified among the six groups (Figure 3A, Table S2). The MS spectra of partial differential metabolites are shown in the Supplementary Figure S3, and the MS fragment information of differential metabolites is shown in Table S3. These 79 differential metabolites were further classified based on their chemical structure and functional groups. As shown in Figure 3B, the majority of differential metabolites belonged to phenylpropanoids and polyketides (26.58%), followed by lipids and lipid-like molecules (21.52%), organic acids and derivatives (16.46%), and organoheterocyclic compounds (16.46%). This distribution suggests that phenylpropanoid-related compounds, which are known to be involved in plant defense and environmental adaptation, represent the most regionally responsive metabolic category. These results also indicate that flavonoids, phenolic acids, and related aromatic compounds play a central role in mediating the biochemical adaptation of mulberry leaves to regional environmental conditions. Compared to the overall composition of all 3794 detected metabolites, phenylpropanoids and polyketides are relatively enriched among the differentially accumulated metabolites, suggesting selective modulation of this class in response to geographic variation. To visually assess the distribution of differential metabolites across the six regions, a heatmap was generated based on their relative abundance (Figure 3C). The heatmap revealed notable differences in the composition of differential metabolites among mulberry leaf samples from the six regions. Cluster analysis demonstrated a clear geographical trend in the grouping of samples. Specifically, the ZY and XJ samples clustered together, indicating a high degree of similarity in their metabolite expression patterns. The NC and NJ samples also clustered together and formed a distinct group separate from ZY-XJ. Notably, the XZ and PZH samples formed a unique branch, exhibiting metabolite features markedly distinct from those of other regions. The XZ-PZH group exhibited a relatively high abundance of flavonoids and organic acids. In contrast, the NC-NJ group was enriched in amino acid derivatives, while the ZY-XJ group showed elevated levels of both amino acid derivatives and flavonoids.

### 3.4. Differential Metabolite Pathway Annotation and Enrichment Analysis

**Figure 4:**
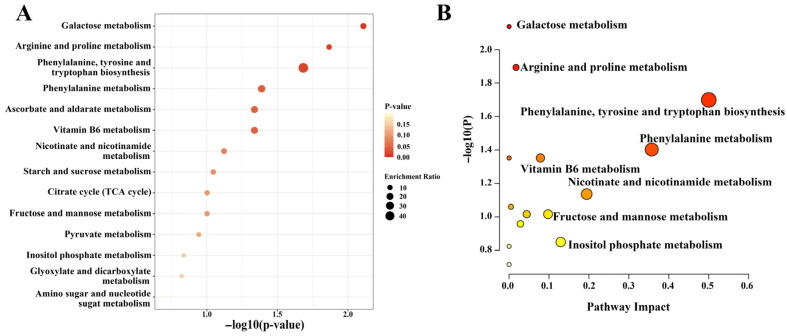
(**A**) KEGG enrichment bubble plot; (**B**) KEGG metabolic pathway impact plot. Red indicates pathways with a high impact (*p* < 0.05), yellow indicates pathways with moderate impact, and white indicates pathways with low impact.

Further analysis was conducted on the key metabolic pathways enriched by the selected differential metabolites. A lower *p*-value indicates a more statistically significant pathway enrichment. As shown in Figure 4A, the 79 significant metabolites identified in mulberry leaf samples from various regions, including XZ, XJ, and PZH, were primarily associated with 14 metabolic pathways. The top three pathways with the most significant enrichment of differential metabolites were galactose metabolism, arginine and proline metabolism, and phenylalanine, tyrosine and tryptophan biosynthesis. KEGG pathway analysis (Figure 4B) revealed significant alterations in several metabolic pathways among the differential metabolites. Notably, galactose metabolism and arginine and proline metabolism pathways exhibited the highest statistical significance, with −log10(P) values approaching 2.0, suggesting their crucial roles in regional variations in mulberry leaf metabolism. Additionally, phenylalanine, tyrosine and tryptophan biosynthesis, along with phenylalanine metabolism, demonstrated high pathway impact scores (close to 0.5), indicating that differential metabolites represent a considerable proportion of the compounds involved in these pathways.

### 3.5. Influence of Key Environmental Factors on Regional Metabolite Variation

**Figure 5:**
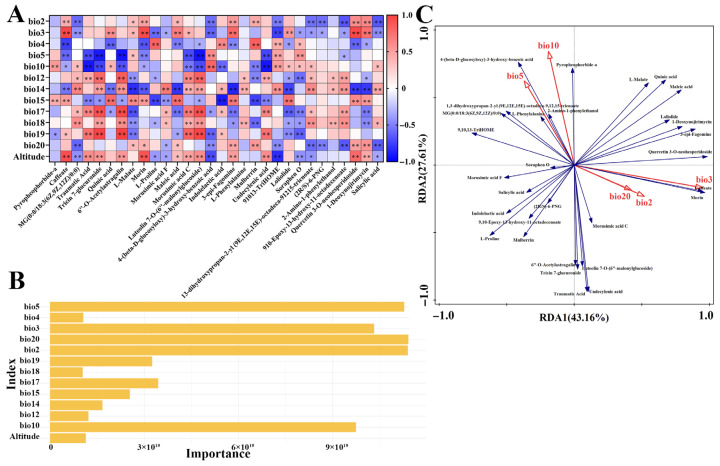
(**A**) Pearson correlation diagram. * indicates *p* < 0.05, ** indicates *p* < 0.01. (**B**) Random Forest diagram. (**C**) Redundancy Analysis (RDA) between differential metabolites and climate factors. The red vectors represent climate factors, and the blue vectors represent differential metabolites. The length of the connecting lines indicates the degree to which the differential metabolites are influenced by the climate factors. The cosine of the angle between the differential metabolites and the climate factors represents the correlation between the two.

A correlation analysis was performed between 30 differential metabolites (ranked by VIP values) and 23 geographical and climatic variables. After selecting the top three associated factors per metabolite and conducting a collinearity test (VIF < 10), 13 key factors were identified: bio2, bio3, bio4, bio5, bio10, bio12, bio14, bio15, bio17, bio18, bio19, bio20, and altitude. Most metabolites showed significant correlations with these environmental factors (Figure 5A). Specifically, flavonoid content was positively correlated with precipitation-related variables (bio12, bio14, bio17, bio18) but negatively correlated with temperature metrics (e.g., bio5, bio10). Phenolic acids were positively linked to precipitation (bio14, bio18) but negatively associated with isothermality (bio3), annual sunshine duration (bio20), and altitude. In contrast, alkaloids exhibited positive correlations with mean monthly temperature range (bio2), isothermality (bio3), annual sunshine duration (bio20), and altitude, and negative associations with temperature seasonality (bio4) and precipitation (bio14, bio17, bio18).

To further quantify environmental contributions, a Random Forest model was applied. It identified bio2, bio3, bio5, bio10, and bio20 as having the strongest explanatory power for metabolite variation (Figure 5B). These variables were subsequently used in an RDA, where the first two axes explained 43.16% and 27.61% of the total variance, respectively. Climatic factors collectively accounted for 70.77% of the variance in metabolite profiles, with individual contributions ranked as: bio3 (40.4%, *p* = 0.002) > bio10 (22.8%, *p* = 0.002) > bio2 (12.7%, *p* = 0.002) > bio5 (11.6%, *p* = 0.002) > bio20 (9.6%, *p* = 0.002). The RDA ordination (Figure 5C) indicated that bio3 strongly influenced Axis 1, while bio10 primarily drove Axis 2. Alkaloids such as 1-deoxynojirimycin and 3-epi-Fagomine showed positive correlations with bio3, bio2, and bio20, whereas certain flavonoids were negatively correlated with bio5 and bio10, and phenolic acids were inversely associated with bio2, bio3, and bio20. These findings align with the Pearson correlation results.

## 4. Discussion

### 4.1. Environmental Regulation of Flavonoid Biosynthesis

Secondary metabolism constitutes a key interface linking environmental conditions to the accumulation of pharmacologically active constituents in plants [23]. The biosynthesis and accumulation of secondary metabolites are profoundly influenced by environmental factors. For instance, increased precipitation promotes flavonoid biosynthesis in species such as *Prunella vulgaris* L. and *Tetrastigma hemsleyanum* Diels & Gilg [24,25]. This observation is consistent with the present study, which revealed a positive correlation between precipitation and flavonoid content. Notably, differential metabolites in mulberry leaves were significantly enriched in phenylalanine, tyrosine, and tryptophan biosynthesis pathways, indicating enhanced aromatic amino acid metabolism. This metabolic activation supplies key precursors, such as phenylalanine, for downstream flavonoid biosynthesis [26]. Supporting this, studies in tea plants have shown that at a relative humidity of 80–85%, key genes in the shikimate pathway (*CsaroDE1-3*) and phenylalanine biosynthesis (*CsPAL*) are upregulated, which synergistically interact with flavonoid biosynthesis genes (*CsCHS*, *CsF3H*, *CsANS*) and ultimately enhancing epigallocatechin gallate (EGCG) accumulation [27]. These findings further underscore the importance of water availability in regulating flavonoid biosynthesis. In addition, humid regions like Medog often experience intermittent UV radiation triggered by cloud cover variations. Intermittent UV exposure may activate the UVR8-MYB signaling cascade, thereby enhancing flavonoid biosynthesis as a photoprotective mechanism [28]. UV-B irradiation of buckwheat sprouts has been shown to rapidly increase the activity of phenylalanine ammonia-lyase (PAL) and chalcone isomerase (CHI), thereby significantly enhancing flavonoid accumulation [29]. Furthermore, multiple studies have confirmed that low temperatures can significantly induce PAL activity in mulberry leaves [30,31]. As the rate-limiting enzyme in the phenylpropanoid pathway, PAL exhibits elevated activity under cold stress, thereby directing greater carbon flux toward flavonoid biosynthesis.

Beyond precursor supply and pathway enrichment, climatic factors likely act through conserved signaling modules converging on flavonoid structural genes. Intermittent UV exposure can activate the UVR8-COP1-HY5 axis, recruit MYB-bHLH-WD40 complexes to upregulate *CHS*, *CHI*, and *F3H*, and thereby enhance epidermal flavonoid accumulation for photoprotection [32]. Cold and large diurnal temperature ranges can increase ROS and trigger MAPK/CBF cascades, thereby inducing upstream phenylpropanoid steps *(PAL*, *C4H*, *4CL*) and boosting carbon flux toward flavonoids [33]. Water availability can further modulate ABA-SnRK2 signaling and downstream ABF/AREB transcription factors, thereby coordinating osmoprotection with late-stage modification (UGTs/OMTs) and vacuolar sequestration (MATE/ABCC transporters) that stabilize and compartmentalize flavonoids [34]. This stimulus-TF-enzyme-metabolite framework provides a coherent mechanistic context for the climate-flavonoid link observed across regions. Based on these hierarchical networks, we propose that such regulation may underlie the marked flavonoid and polyphenol enrichment observed in mulberry leaves from XZ (Tibet). The persistent humidity, cold and intermittent UV radiation in this mountainous region likely contribute to the enhanced accumulation of these bioactive compounds as part of an adaptive defense strategy.

### 4.2. Photothermal Coupling and Alkaloid Accumulation

Similarly, alkaloids as another important class of secondary metabolites are also regulated by various environmental factors in their synthesis and accumulation. Empirical evidence indicates that DNJ content in mulberry leaves follows a bell-shaped temporal distribution, peaking in mid-August and strongly correlating with cumulative temperature [35]. Notably, exposure to frost stress leads to a sharp decline in DNJ concentrations in mulberry leaves [30]. A study conducted in the mountainous regions of central Guizhou revealed that larger diurnal temperature fluctuations were positively associated with increased levels of (−)-Anabasine in tobacco [36]. In *Camptotheca acuminata* seedlings, incubation at 40 °C for 2 h resulted in a 6-fold increase in hydroxycamptothecin accumulation [37]. Field-grown cultivars of *Lupinus angustifolius* exposed to elevated temperatures exhibited significantly enhanced alkaloid accumulation [38]. Conversely, exposure to low temperatures significantly reduces the levels of vindoline and catharanthine in *Catharanthus roseus* leaves [39].

Transcriptomic analysis demonstrated that prolonged high-temperature exposure significantly induced the expression of lysine decarboxylase (LDC) and downstream genes including *CYP450* and methyltransferase (MT), thereby enhancing DNJ biosynthesis in mulberry [40]. Another study identified dehydrogenase MnGutB1 as a key enzyme in glucose-derived DNJ biosynthetic pathway. Under high-temperature and long-day conditions, sterile seedlings exhibited significantly elevated MnGutB1 transcript levels compared to controls, resulting in a marked increase in DNJ content [41].

Collectively, these findings demonstrate that key enzymes involved in both glucose- and lysine-derived DNJ biosynthetic pathways are coordinately upregulated under high-temperature long-day conditions, providing multi-level evidence for photothermal coupling-mediated DNJ accumulation. This mechanistic framework may explain the high DNJ contents in mulberry leaves from PZH and NC, as both regions exhibit photothermal conditions compatible with DNJ biosynthesis. PZH, which has the highest DNJ content (3.65 ± 0.03 mg/g), exhibits a mean annual temperature of 21.6–22.0 °C, a warmest-month maximum of 34.6–40.5 °C, and an annual sunshine duration of 2668.9–2816.4 h, respectively. Notably, the difference in DNJ content between PZH and NC (3.65 mg/g vs. 1.99 mg/g) is consistent with a putative association between photothermal intensity and DNJ biosynthesis. Stronger photothermal conditions (higher temperature and longer sunshine duration) may more strongly induce key enzymes in both DNJ synthetic pathways and may thereby increase DNJ accumulation. This observation further supports the view that photothermal coupling may be a core regulatory factor for DNJ accumulation in mulberry leaves.

### 4.3. Metabolic Variations in Plant Adaptation Driven by Environmental Factors

Metabolomic profiling revealed distinct differences in mulberry leaf metabolites across regions with contrasting climatic conditions, particularly in Medog (XZ) and Panzhihua (PZH). While the 2023–2024 sampling timeframe limited long-term trend analysis, the data clearly demonstrated that regional environmental factors significantly influenced the accumulation of key bioactive compounds.

As the region with the highest precipitation in Tibet (annual precipitation of 2000–3500 mm, with over 200 rainy days annually), Medog has persistently high air humidity (relative humidity often exceeding 80%). In this high-humidity environment, plants remain moist for extended periods, making them susceptible to fungal and bacterial infections [42,43]. Flavonoids and polyphenols, as antimicrobial metabolites, play an important role in protecting plants from biological stress [44]. Therefore, plants may actively accumulate high levels of flavonoids and phenolic compounds to develop adaptive defense mechanisms against fungal and bacterial infections received in high humidity environments [45,46,47]. In addition, the difference in regional temperature and sunshine further enhanced the regional differentiation of metabolites. PZH, belonging to the South Subtropical Dry Hot Valley climate (annual precipitation of only 700–1000 mm), has low risks of pathogen proliferation. Consequently, plants do not require extensive synthesis of such “defensive metabolites”. However, high temperature and strong sunlight lead to increased levels of active oxygen, increasing the risk of oxidative stress [48,49]. This makes the antioxidant functions of flavonoids and polyphenols particularly important. Plants need to synthesize these compounds to remove active oxygen and reduce oxidative damage. They can also absorb ultraviolet rays, reduce light damage and enhance light protection ability. The longer sunlight hours in PZH improve photosynthetic efficiency, providing enough substrates for alkaloid synthesis. In this environment, plants may simultaneously synthesize alkaloids, flavonoids, and polyphenol compounds through coordinated metabolic flow regulation. This optimizes the metabolic network, improves resource-utilization efficiency and achieves efficient metabolite accumulation to adapt to the dry valley climate. This comprehensive metabolic regulation mechanism reflects the adaptive strategies of plants.

Although the interactive mechanisms among factors like temperature, light, and water availability require further dissection. This geographic-specific metabolic profiling provides a foundational dataset for optimizing cultivation strategies to enhance the content of desired bioactive constituents.

### 4.4. Stress-Responsive Metabolic Pathways and Their Environmental Inducers

Pathway enrichment indicated that several pathways—galactose metabolism, phenylalanine, tyrosine and tryptophan biosynthesis, and arginine and proline metabolism—are closely linked to plant responses to abiotic stress. These pathways likely reflect region-specific metabolic adaptations to distinct environmental pressures. For example, galactose metabolism contributes to the biosynthesis of raffinose family oligosaccharides (RFOs), which function as osmoprotectants under drought and high-light conditions [50]. Enrichment of this pathway in PZH may reflect the arid climate and intense solar radiation characteristic of this dry-hot valley region. Biosynthesis of aromatic amino acids (phenylalanine, tyrosine, and tryptophan) provides key precursors for stress-related secondary metabolites, such as flavonoids and alkaloids. These pathways are often activated by UV exposure and temperature fluctuations [51,52], factors prevalent in XZ owing to high-altitude UV radiation and cold stress. Arginine and proline metabolism also plays a central role in mitigating oxidative stress, as proline accumulation enhances osmotic balance and scavenges ROS [53]. Enrichment in regions with large diurnal temperature ranges, such as XJ and PZH, may represent an adaptive mechanism to maintain cellular homeostasis. Collectively, these observations suggest that precipitation, UV radiation, and temperature are key stressors shaping regional metabolic profiles in mulberry leaves. They also provide mechanistic insight into stress-responsive metabolic reprogramming.

### 4.5. Regional Heterogeneity and Genetic Variation in Mulberry Leaf Secondary Metabolites

This study identified marked regional heterogeneity in mulberry leaf chemistry based on quantitative measurements of total flavonoids, total polyphenols, and DNJ content across six regions. Among the six regions, XZ had the highest contents of total flavonoids (71.94 ± 2.04 mg/g) and total polyphenols (30.63 ± 0.41 mg/g). By contrast, DNJ levels were relatively higher in PZH and NC (3.65 ± 0.03 mg/g and 1.99 ± 0.17 mg/g, respectively). These findings suggest that regional environmental heterogeneity—spanning climate, soil, and altitude—may regulate the accumulation of these bioactive constituents in mulberry leaves. Comparison with prior reports further supports the geographic dependence of mulberry leaf chemical composition. For instance, Deng reported total polyphenols of 282.50–394.43 mg/g and total flavonoids of 21.30–43.02 mg/g in leaves from eight regions [54], whereas Guan reported DNJ levels of 8.652–10.392 mg/g in leaves from three production areas [55]. Together, these studies revealed significant inter-regional differences in target constituents, consistent with our results. Collectively, these data support a substantial influence of geographical factors on the accumulation of bioactive constituents in mulberry leaves.

Beyond environmental drivers, genetic differences among mulberry populations may also contribute materially to the observed chemical variation. *M. alba* L. is widely cultivated worldwide, and regional populations are likely to have accrued genetic divergence through long-term geographic isolation and local adaptation. Such genetic variations can directly affect the biosynthesis and accumulation of secondary metabolites. Previous studies show that mulberries with different genetic backgrounds exhibit marked differences in the types and contents of these metabolites [56,57], and that genetic factors often act synergistically with environmental conditions to modulate metabolic pathways. Notably, although all samples were collected within China during the same growing season, regional populations may still possess distinct genetic backgrounds, which could partially explain interregional variation in TFC, TPC, and DNJ levels. However, because no systematic genotypic was performed, the specific contribution of genetic differences to the observed metabolic variation cannot be resolved here. Future work should integrate genotyping (e.g., SNP or transcriptomic profiling) with environmental data to clarify the mechanisms underlying variations in mulberry leaf secondary metabolites.

## 5. Conclusions

This study shows that the chemical composition of mulberry leaves is significantly influenced by their growing environment. Quantitative analysis of total flavonoids, total polyphenols, and DNJ across six Chinese regions showed that samples from Tibet (XZ) had the highest total flavonoids and total polyphenols, whereas samples from Panzhihua (PZH) had the highest DNJ levels. Untargeted metabolomics identified 3794 metabolites, of which 79 differential metabolites were significantly enriched in galactose pathways metabolism, phenylalanine biosynthesis, and arginine and proline metabolism. These pathways are closely associated with plant stress responses and secondary-metabolite synthesis. Climate analysis indicated that temperature (e.g., isothermality, mean temperature of the warmest quarter) and light (annual sunshine duration) are the main environmental drivers. Together, these results underscore the central role of climate in shaping mulberry leaf chemistry. Climate regimes shape distinct metabolic profiles; high-altitude humid conditions tend to promote flavonoid and polyphenol accumulation, whereas warm conditions with prolonged sunlight favor DNJ biosynthesis.

These findings provide a scientific basis to inform quality evaluation and the selection of optimal cultivation regions for mulberry leaves. Future research should integrate genetic analysis (e.g., genotyping or transcriptomics) with environmental data to clarify the mechanisms underlying variations in mulberry leaf secondary metabolites, thereby further elucidating the interplay between genetic and environmental influences on plant metabolism.

## Figures and Tables

**Table 1:** TFC, TPC, and DNJ Contents in Mulberry Leaves from Different Regions (mg/g).

Sample ID	TFC	TPC	DNJ
NC	14.46 ± 0.58 ^e^	6.61 ± 0.54 ^e^	1.99 ± 0.17 ^b^
NJ	12.99 ± 0.55 ^e^	8.29 ± 0.01 ^cde^	0.75 ± 0.11 ^c^
PZH	41.13 ± 0.40 ^b^	15.90 ± 0.37 ^b^	3.65 ± 0.03 ^a^
ZY	30.50 ± 1.18 ^c^	14.65 ± 2.67 ^abcde^	0.45 ± 0.07 ^d^
XJ	19.86 ± 0.47 ^d^	10.24 ± 0.45 ^cd^	0.80 ± 0.05 ^c^
XZ	71.94 ± 2.04 ^a^	30.63 ± 0.41 ^a^	0.14 ± 0.01 ^e^

Note: The data are expressed as Mean ± SD. One-way ANOVA followed by Duncan’s multiple range test was performed for each column. Different lowercase letters in the same column indicate significant differences among groups (*p* < 0.05).

## Data Availability

The original contributions presented in this study are included in the article/Supplementary Material. Further inquiries can be directed to the corresponding author.

## Supplementary Materials

The following supporting information can be downloaded at: https://www.mdpi.com/article/10.3390/metabo15110728/s1, Figure S1: Total ion flow chromatogram of samples; Figure S2: Total ion flow chromatogram of QC; Figure S3: Partial differential metabolites MS mass spectra; Table S1: Geographical and climatic factors at six sampling sites in 2023–2024; Table S2: Differential metabolites in mulberry leaves; Table S3: MS fragmentations for differential metabolites in mulberry leaves.
