# Supplementary material for: Regional Variation in Mulberry Leaf Metabolites: A Combined Metabolomic and Environmental Analysis of Biosynthetic Drivers
Source: Metabolites. 2025 Nov 6;15(11):728. doi: 10.3390/metabo15110728 (PMC12654259; doi:10.3390/metabo15110728)
Supplement: Supplementary file 1 [file metabolites-15-00728-s001.zip › Supplementary Materials—— Summary of Figures (with Captions).pdf]

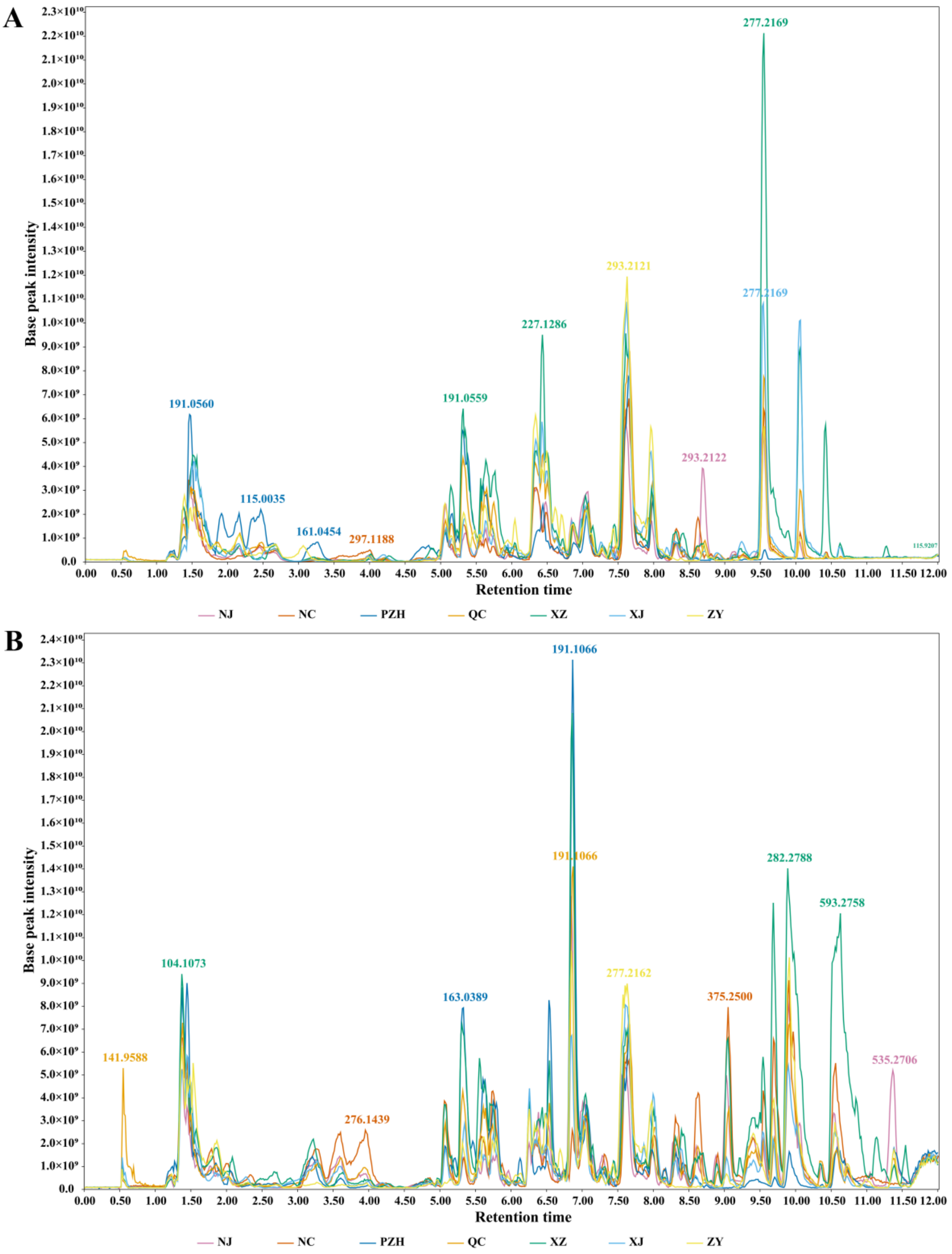

Supplementary Figure S1. Total ion flow chromatogram of samples. (**A**): Negative ion mode; (**B**): Positive ion mode.

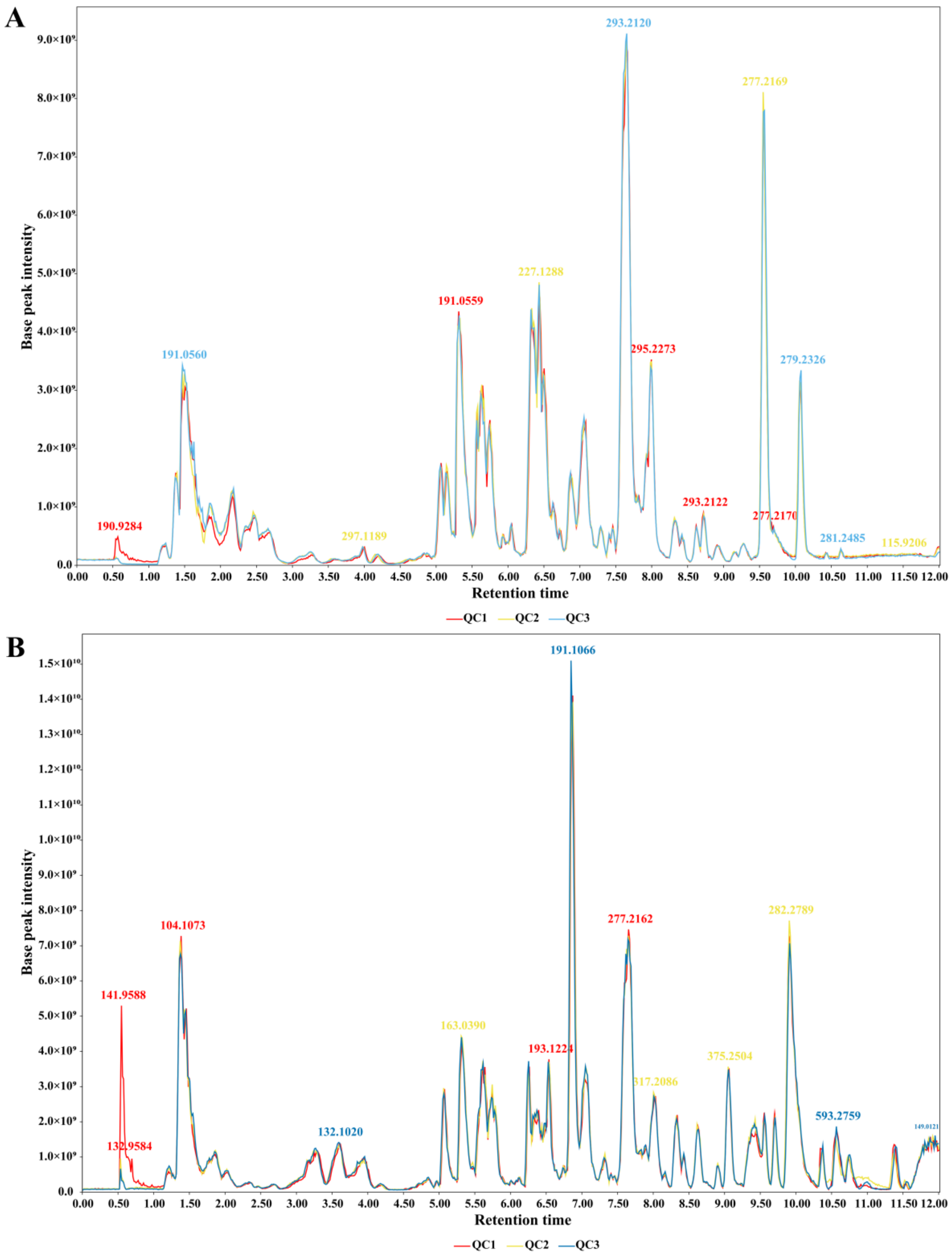

Supplementary Figure S2. Total ion flow chromatogram of QC. (**A**): Negative ion mode; (**B**): Positive ion mode.

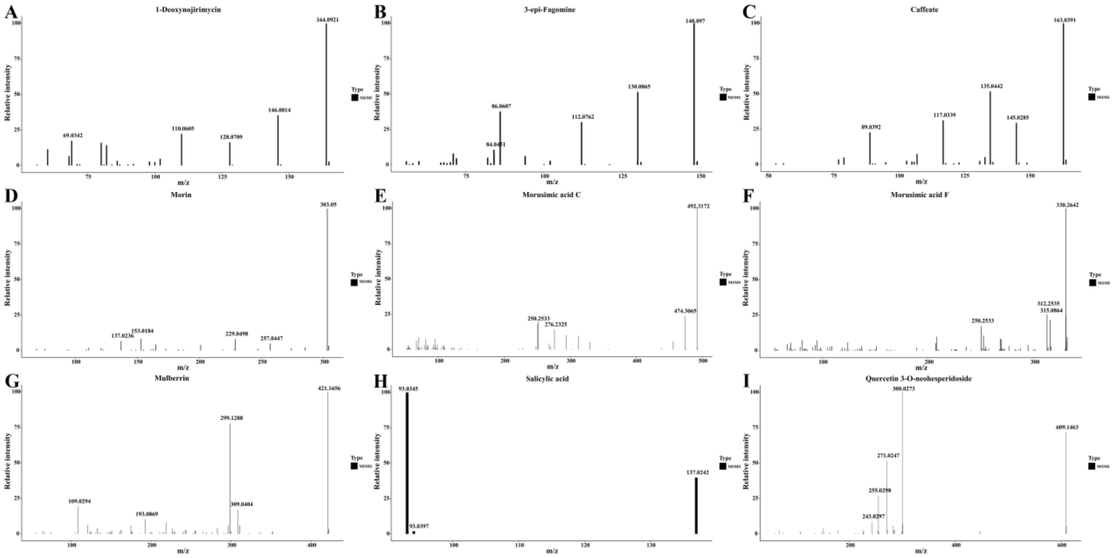

Supplementary Figure S3. Partial differential metabolites MS mass spectra. (**A**): 1-Deoxynojirimycin; (**B**): 3-epi-Fagomine; (**C**): Caffeate; (**D**): Morin; (**E**): Morusimic acid C; (**F**): Morusimic acid F; (**G**): Mulberrin; (**H**): Salicylic acid; (**I**): Quercetin 3-O-neohesperidoside.
