# Supplementary material for: Regional Variation in Mulberry Leaf Metabolites: A Combined Metabolomic and Environmental Analysis of Biosynthetic Drivers
Source: Metabolites. 2025 Nov 6;15(11):728. doi: 10.3390/metabo15110728 (PMC12654259; doi:10.3390/metabo15110728)
Supplement: Supplementary file 1 [file metabolites-15-00728-s001.zip › Supplementary Table S1.pdf]

Table S1 Geographical and climatic factors at six sampling sites in 2023–2024

Climatic fcator	Interpretation	NC		NJ		PZH		ZY		XJ		XZ	
		2023	2024	2023	2024	2023	2024	2023	2024	2023	2024	2023	2024
bio1	Mean annual temperature	18.1	19	19.4	19.8	22	21.6	18.4	18.9	12	11.8	16.7	17
bio2	Mean monthly temperature range	7.9	7.6	7.3	6.9	12.2	12	7.3	7	12.5	12.1	8.3	8.2
bio3	Isothermality	20.2	18.6	18.6	17	30.1	34.7	18.6	17.4	19	21.7	26.5	25.4
bio4	Temperature seasonality	711	809	704	774	469	480	718	800	1159	1131	462	522
bio5	Max temperature of warmest month	37.6	40.6	39	40.7	40.5	34.6	38.2	40.2	39.6	39.8	31.3	32.3
bio6	Min temperature of coldest month	−1.5	−0.2	−0.3	0	0	0	−1.1	0	−26.2	−15.9	0	0
bio7	Temperature annual range	39.1	40.8	39.3	40.7	40.5	34.6	39.3	40.2	65.8	55.7	31.3	32.3
bio8	Mean temperature of wettest quarter	26.7	29.5	27.6	29.8	25.6	25.6	26.8	29.2	5.9	3.9	22.7	18.8
bio9	Mean temperature of driest quarter	10.2	10.5	11.6	11.8	17.8	18.3	10.6	10.6	23.6	23.2	11.8	10.8
bio10	Mean temperature of warmest quarter	26.7	29.5	27.6	29.8	26.7	25.8	26.8	29.2	23.6	23.2	22.7	23.7
bio11	Mean temperature of coldest quarter	10.2	10.5	11.6	11.8	17.8	16.7	10.6	10.6	−0.6	−0.1	11.8	10.8
bio12	Annual precipitation	1084	922.9	764	1066.4	461	835.7	696.3	834.8	245.8	289.7	2186.1	1963.8
bio13	Precipitation of wettest month	282.4	250.9	249.1	324.1	189.7	228.9	186.3	277.8	43.8	38.2	371.9	449.2
bio14	Precipitation	1.6	9.1	0.5	6.6	0	0	1.4	6	0.1	6.6	3.7	7.3

	of driest month												
bio15	Precipitation seasonality/(Coefficient of Variation)	89	84	108	98	142	118	99	108	59	38	71	90
bio16	Precipitation of wettest quarter	619.9	447.5	477.9	501.8	268.7	486.5	445.8	380.1	92.1	91	884.1	926.3
bio17	Precipitation of direst quarter	83.3	62.5	32.1	56.8	7	0	31.1	50.3	38.9	51.7	217	131.5
bio18	Precipitation of warmest quarter	619.9	447.5	477.9	501.8	121.8	251	445.8	380.1	38.9	51.7	884.1	589
bio19	Precipitation of coldest quarter	83.3	62.5	32.1	56.8	7	98.2	31.1	50.3	68.4	61.4	217	131.5
bio20	Annual sunshine duration	1360.1	1327.4	1280.5	1325.7	2816.4	2668.9	1238.5	1408.4	3105.6	2968	1449.3	1326.1
Longitude	Longitude	106.1319		104.8536		101.7503		105.03		80.8458		95.3175	
Latitude	Latitude	30.7456		29.7678		26.495		30.2808		44.0489		29.3128	
Altitude	Altitude	347		369.3		1280.6		462.6		624.3		1305	
