# Supplementary material for: Regional Variation in Mulberry Leaf Metabolites: A Combined Metabolomic and Environmental Analysis of Biosynthetic Drivers
Source: Metabolites. 2025 Nov 6;15(11):728. doi: 10.3390/metabo15110728 (PMC12654259; doi:10.3390/metabo15110728)
Supplement: Supplementary file 1 [file metabolites-15-00728-s001.zip › Supplementary Table S2.pdf]

Table S2 Differential metabolites in mulberry leaves

Name	VIP	P value				
		NC vs XZ	NJ vs XZ	PZH vs XZ	XJ vs XZ	ZY vs XZ
(2R/S)-6-PNG	3.94886	9.71×10^{-11}	1.39×10^{-5}	1.25×10^{-10}	3.69×10^{-10}	5.76×10^{-8}
(R)-8-Methylsulfinyloctyl isothiocyanate	2.1337	2.26×10^{-9}	2.79×10^{-6}	1.07×10^{-6}	5.25×10^{-9}	1.89×10^{-6}
1,3-dihydroxypropan-2-yl (9E,12E,15E)-octadeca-9,12,15-trienoate	4.00516	5.06×10^{-4}	2.59×10^{-4}	1.20×10^{-8}	1.51×10^{-7}	5.21×10^{-9}
10-Deacetoxybarbilycopodin	2.22046	3.64×10^{-4}	3.30×10^{-9}	6.96×10^{-6}	1.72×10^{-11}	7.15×10^{-8}
13-OxoODE	3.13189	1.18×10^{-3}	3.80×10^{-3}	2.07×10^{-4}	8.18×10^{-12}	1.28×10^{-9}
1-Deoxynojirimycin	3.83704	2.63×10^{-12}	1.79×10^{-3}	7.69×10^{-7}	1.59×10^{-9}	4.29×10^{-5}
2-Amino-1-phenylethanol	3.89238	2.11×10^{-6}	8.86×10^{-3}	1.20×10^{-5}	2.39×10^{-7}	3.44×10^{-11}
2'-Hydroxygenistein 7-O-(6''-malonylglucoside)	2.63608	6.18×10^{-13}	8.04×10^{-14}	5.89×10^{-14}	1.12×10^{-12}	1.01×10^{-13}
2-Hydroxymethyl benzoic acid	2.14756	1.11×10^{-8}	9.28×10^{-9}	1.82×10^{-3}	1.23×10^{-8}	1.00×10^{-7}
2-Palmitoylglycerol	2.50559	3.67×10-^{3}	9.52×10^{-3}	7.53×10^{-6}	6.28×10^{-4}	1.58×10^{-5}
3-[(2-Oxoacetyl)oxy]-4-(trimethylazaniumyl)butanoate	2.83527	3.90×10^{-6}	2.24×10^{-4}	1.37×10^{-7}	4.54×10^{-3}	5.21×10^{-5}
3-epi-Fagomine	4.49387	8.60×10^{-12}	1.00×10^{-4}	1.33×10^{-9}	2.18×10^{-7}	2.70×10^{-13}
4-(beta-D-glucosyloxy)-3-hydroxy-benzoic acid	4.83234	2.94×10^{-10}	1.26×10^{-11}	3.61×10^{-6}	8.80×10^{-9}	5.09×10^{-12}
4-guanidinobutanoate	2.0508	1.04×10^{-9}	5.79×10^{-8}	1.33×10^{-4}	2.41×10^{-3}	3.13×10^{-9}
4-n-Pentylphenol	2.44666	2.99×10^{-11}	2.25×10^{-8}	1.77×10^{-8}	1.78×10^{-4}	3.05×10^{-8}
6''-O-Acetylastragalin	6.08172	1.87×10^{-13}	2.87×10^{-14}	2.22×10^{-14}	2.31×10^{-13}	3.69×10^{-14}
9(S)-HpOTrE	3.60278	7.08×10^{-9}	9.56×10^{-9}	3.88×10^{-6}	1.25×10^{-8}	1.21×10^{-11}
9,10,13-TriHOME	4.10619	1.45×10^{-10}	4.22×10^{-5}	9.86×10^{-9}	5.15×10^{-10}	7.09×10^{-11}
9,10-Epoxy-13-hydroxy-11-octadecenoate	3.88274	1.26×10^{-5}	2.63×10^{-5}	2.97×10^{-8}	1.37 × 10-3	2.18×10^{-2}
Albocycline M-6	2.25762	1.05×10^{-5}	3.75×10^{-11}	7.99×10^{-13}	1.37×10^{-13}	7.02×10^{-12}
alpha-Cyclogeraniol acetate	2.40396	1.37×10^{-7}	1.21×10^{-3}	4.94×10^{-2}	1.09×10^{-8}	2.29×10^{-11}
alpha-Isopropylmalate	2.77973	2.33×10^{-12}	1.04×10^{-14}	4.11×10^{-8}	2.53×10^{-5}	1.35×10^{-10}
Baimaside	2.05153	4.41×10^{-12}	4.59×10^{-10}	4.28×10^{-8}	1.30×10^{-11}	2.03×10^{-9}
Broussonetine F	2.02622	3.32×10^{-8}	2.17×10^{-7}	4.80×10^{-11}	1.14×10^{-3}	7.47×10^{-9}
Butylidenephthalide	2.5271	3.29×10^{-7}	7.19×10^{-3}	3.07×10^{-8}	5.62×10^{-5}	4.11×10^{-5}
Caffeate	7.09753	1.01×10^{-7}	7.62×10^{-8}	1.47×10^{-4}	1.18×10^{-7}	6.67×10^{-7}
CARBETAPENTANE	2.03051	6.65×10^{-12}	7.76×10^{-12}	1.50×10^{-11}	1.20×10^{-5}	3.61×10^{-6}
Compactin diol lactone	2.39271	5.18×10^{-3}	1.24×10^{-4}	2.74×10^{-4}	1.04×10^{-10}	1.54×10^{-13}
D(+)-Raffinose	2.00031	2.08×10^{-11}	3.58×10^{-10}	9.68×10^{-12}	2.04 × 10-7	7.00×10^{-13}
D-Fructose	2.57501	6.06×10^{-7}	8.79×10^{-12}	8.47×10^{-12}	1.14×10^{-11}	9.41×10^{-12}
DGMG 18:3	3.0182	5.15×10^{-7}	4.50×10^{-4}	3.54×10^{-2}	2.21×10^{-4}	1.57×10^{-13}
Dibutyl maleate	3.56875	7.23×10^{-9}	1.38×10^{-8}	9.10×10^{-7}	6.58×10^{-4}	3.68×10^{-7}
Dichotosinin	2.42081	2.65×10^{-10}	8.68×10^{-13}	1.68×10^{-8}	1.54×10^{-12}	6.70×10^{-7}
Esculetin	2.61833	7.42×10^{-3}	1.52×10^{-3}	1.19×10^{-5}	2.58×10^{-4}	1.71×10^{-10}
Fabianine	2.32194	9.45×10^{-12}	7.64×10^{-12}	6.31×10^{-11}	1.36×10^{-10}	3.91×10^{-5}
Gastrodin	2.37469	1.11×10^{-4}	2.94×10^{-2}	3.81×10^{-4}	1.15×10^{-7}	3.82×10^{-12}
Indolelactic acid	4.63828	1.24×10^{-5}	2.32×10^{-3}	6.60×10^{-11}	3.60×10^{-8}	2.15×10^{-11}
Inositol	2.50529	5.83×10^{-8}	2.48×10^{-9}	8.90×10^{-9}	1.25×10^{-12}	8.83×10^{-7}
Isoscopoletin	3.24517	1.21×10^{-5}	1.20×10^{-3}	7.89×10^{-11}	4.39×10^{-8}	1.96×10^{-8}
L-Malate	6.01277	3.34×10^{-2}	1.91×10-^{6}	1.95×10^{-6}	4.03×10^{-3}	1.38×10^{-4}

Loliolide	4.08398	4.37×10^{-9}	6.63×10^{-3}	9.34×10^{-13}	9.30×10^{-5}	3.29×10^{-7}
L-Phenylalanine	4.44389	8.73×10^{-7}	1.43×10^{-2}	4.33×10^{-6}	5.71×10^{-7}	4.70×10^{-11}
L-Proline	5.48908	2.96×10^{-3}	1.47×10^{-3}	7.85×10^{-10}	2.18×10^{-10}	8.34×10^{-9}
Luteolin 7-O-(6''-malonylglucoside)	5.11126	7.00×10^{-9}	4.34×10^{-9}	4.50×10^{-9}	3.14×10^{-8}	6.39×10^{-9}
Maleic acid	5.42029	1.05×10^{-10}	7.97×10^{-15}	1.15×10^{-10}	1.72×10^{-3}	1.06×10^{-9}
Maryal	2.26387	1.94×10^{-12}	1.26×10^{-9}	2.22×10^{-5}	1.86×10^{-10}	3.47×10^{-14}
Methyl pentadecanoate	3.27292	1.11×10^{-2}	9.87×10^{-3}	6.45×10^{-3}	1.13 × 10-2	8.49×10^{-3}
MG(0:0/18:3(6Z,9Z,12Z)/0:0)	6.68245	1.98×10^{-4}	8.92×10^{-5}	3.26×10^{-12}	4.02×10^{-9}	3.61×10^{-11}
Moracin N	2.52989	9.29×10^{-5}	1.82×10^{-2}	2.64×10^{-4}	5.55×10^{-3}	2.14×10^{-6}
Morin	5.64766	6.60×10^{-9}	3.15×10^{-8}	8.67×10^{-7}	2.47×10^{-7}	4.46×10^{-6}
Morusimic acid C	5.40856	2.77×10^{-3}	2.01×10^{-10}	2.66×10^{-8}	1.28×10^{-8}	4.50×10^{-11}
Morusimic acid F	5.43482	2.83×10^{-7}	8.26×10^{-7}	6.88×10^{-9}	2.84×10^{-2}	1.22×10^{-4}
Mulberrin	4.12969	1.65×10^{-6}	6.86×10^{-4}	6.45×10^{-7}	4.12×10^{-10}	4.03×10^{-7}
N-acetylphenylalanine	2.41827	7.15×10^{-9}	6.02×10^{-17}	4.39 × 10-7	4.04×10^{-10}	1.67×10^{-11}
N-decanoylglycine	2.68556	3.42×10^{-9}	4.64×10^{-10}	5.95×10^{-15}	6.47×10^{-12}	8.75×10^{-10}
N-Fructosyl tyrosine	2.71732	3.76×10^{-2}	1.44×10^{-9}	5.98×10^{-10}	3.29×10^{-9}	1.04×10^{-9}
Nicotiflorin	2.96931	4.04×10^{-6}	4.05×10^{-5}	3.10×10^{-12}	7.60×10^{-5}	1.92×10^{-9}
Nicotinamide	2.5436	1.67×10^{-10}	1.39×10^{-10}	3.09×10^{-2}	2.30×10^{-10}	6.23×10^{-11}
O-methylmalonylcarnitine	3.68335	1.19×10^{-6}	2.19×10^{-3}	6.35×10^{-6}	2.58×10^{-2}	1.99×10^{-5}
O-Succinyhomoserine	3.26952	1.94×10^{-5}	4.84×10^{-4}	3.07×10^{-2}	1.06×10^{-3}	1.96×10^{-9}
Pandangolide 1	2.32081	1.50×10^{-11}	3.28×10^{-14}	2.41×10^{-3}	1.81×10^{-14}	3.45×10^{-13}
Perillyl alcohol	2.48168	4.52×10^{-5}	1.73×10^{-3}	2.82×10^{-7}	1.18×10^{-7}	2.65×10^{-5}
Phaeophorbide b	3.3301	1.04×10^{-3}	2.11×10^{-5}	1.11×10^{-3}	8.31×10^{-4}	6.88×10^{-5}
Picolinic acid	2.53272	2.19×10^{-8}	4.01×10^{-8}	4.61×10^{-2}	3.64×10^{-10}	5.81×10^{-11}
Pseudotropine	3.04096	3.24×10^{-7}	1.40×10^{-2}	4.73×10^{-11}	1.86×10^{-9}	2.15×10^{-7}
Pyridoxine	2.04792	5.29×10^{-6}	9.66×10^{-6}	2.78×10^{-4}	2.55×10^{-4}	1.44×10^{-2}
Pyropheophorbide-a	9.86751	3.55×10^{-2}	1.53×10^{-7}	6.08×10^{-4}	2.23×10^{-2}	2.35×10^{-4}
Quercetin 3-O-neohesperidoside	3.86775	4.01×10^{-10}	1.83×10^{-9}	7.23×10^{-11}	3.93×10^{-8}	1.62×10^{-10}
Quinic acid	6.24098	1.07×10^{-9}	9.85×10^{-9}	4.97×10^{-9}	2.40×10^{-12}	1.16×10^{-8}
Rhodiosin	2.09994	3.49×10^{-9}	5.58×10^{-7}	8.00×10^{-9}	1.64×10^{-8}	4.52×10^{-9}
Rosmarinic acid	2.38759	1.76×10^{-16}	2.95×10^{-16}	3.47×10^{-2}	2.77×10^{-16}	6.52×10^{-16}
Salicylic acid	3.73371	5.50×10^{-7}	2.57×10^{-11}	2.53×10^{-12}	8.14×10^{-14}	7.56×10^{-15}
Soraphen O	4.05689	1.20×10^{-11}	5.95×10^{-12}	2.45×10^{-10}	7.47×10^{-14}	1.22×10^{-16}
spinosyn macrolactone	2.77574	1.27×10^{-9}	1.65×10^{-9}	3.81×10^{-6}	7.04×10^{-8}	4.33×10^{-10}
Tetracentronside B	2.54765	1.19×10^{-10}	3.91×10^{-14}	3.19×10^{-6}	4.55×10^{-13}	1.07×10^{-9}
Traumatic Acid	6.65253	1.49×10^{-18}	1.80×10^{-18}	2.56×10^{-15}	1.44×10^{-11}	2.51×10^{-16}
Tricin 7-glucuronide	6.44676	1.35×10^{-7}	3.03×10^{-8}	3.77×10^{-8}	5.81×10^{-7}	4.90×10^{-8}
Umbelliferone	2.18676	1.25×10^{-3}	1.44×10^{-2}	1.29×10^{-8}	5.58×10^{-9}	7.14×10^{-12}
Undecylenic acid	4.11651	9.21×10^{-19}	9.56×10^{-19}	4.24×10^{-15}	1.21×10^{-11}	1.40×10^{-16}
