# Supplementary material for: Regional Variation in Mulberry Leaf Metabolites: A Combined Metabolomic and Environmental Analysis of Biosynthetic Drivers
Source: Metabolites. 2025 Nov 6;15(11):728. doi: 10.3390/metabo15110728 (PMC12654259; doi:10.3390/metabo15110728)
Supplement: Supplementary file 1 [file metabolites-15-00728-s001.zip › Supplementary Table S3.pdf]

Table S3 MS fragmentations for differential metabolites in mulberry leaves

Name	Molecular Weight	m/z	MassError	Adduct	RT (min)	Score	Level
(2R/S)-6-PNG	340.13108	339.1237112	0.20219249	[M-H]-	6.719	0.614991442610376	1
(R)-8-Methylsulfinyloctyl isothiocyanate	233.09081	234.0972836	3.545291657	[M+H]+	1.417	0.505595264872845	2
1,3-dihydroxypropan-2-yl (9E,12E,15E)-octadeca-9,12,15-trienoate	352.26136	353.2685336	0.358881496	[M+H]+	9.052	0.967997373108915	2
10-Deacetoxybarbilycopodin	364.26136	365.2685558	0.285923723	[M+H]+	7.965	0.854339782397632	2
13-OxoODE	294.21949	295.2265003	0.98480429	[M+H]+	7.993	0.942738467621475	2
1-Deoxynojirimycin	163.08446	164.0919311	1.048891912	[M+H]+	1.344	0.975745522666832	1
2-Amino-1-phenylethanol	137.08406	120.0809947	1.712077444	[M+H-H2O]+	5.084	0.999665173892552	1
2'-Hydroxygenistein 7-O-(6"-malonylglucoside)	534.10096	533.0940992	0.822352401	[M-H]-	5.757	0.887714953545967	2
2-Hydroxymethyl benzoic acid	152.04735	135.0442373	1.231988989	[M+H-H2O]+	5.337	0.716095451399733	1
2-Palmitoylglycerol	330.27701	353.2661553	0.165565305	[M+Na]+	9.894	0.985324905360214	1
3-[(2-Oxoacetyl)oxy]-4-(trimethylazaniumyl)butanoate	217.09502	218.1025771	1.184387698	[M+H]+	2.698	-	3
3-epi-Fagomine	147.08954	148.0968447	0.031655902	[M+H]+	1.359	0.588193367429607	2
4-(beta-D-glucosyloxy)-3-hydroxy-benzoic acid	316.07943	315.0722845	0.488885126	[M-H]-	5.073	0.924688641971242	2
4-guanidinobutanoate	145.08513	146.092421	0.062316062	[M+H]+	1.888	0.904017039220625	1
4-n-Pentylphenol	164.12011	165.1275377	0.777894884	[M+H]+	6.436	0.969453831478883	2
6"-O-Acetylastragalin	490.11113	489.1041676	0.688752596	[M-H]-	5.756	0.930138875015291	2
9(S)-HpOTrE	310.21441	309.2071144	0.014313023	[M-H]-	6.583	0.790782204942321	2
9,10,13-TriHOME	330.24063	329.2331039	0.684547129	[M-H]-	6.495	0.903440228759272	2
9,10-Epoxy-13-hydroxy-11-octadecenoate	312.23006	311.2227481	0.038107471	[M-H]-	7.277	0.911597722293207	2
Albocycline M-6	326.20932	325.2023451	0.996557554	[M-H]-	6.567	0.870228056785224	2
alpha-Cyclogeraniol acetate	210.16198	211.1694066	0.602473145	[M+H]+	5.876	0.937139118710441	2
alpha-Isopropylmalate	176.06848	175.0610687	0.632101254	[M-H]-	5.562	0.983585485700547	1
Baimaside	626.14831	625.1416703	1.05456665	[M-H]-	5.489	0.969927415374087	1
Broussonetine F	361.24644	362.2537851	0.124975775	[M+H]+	5.346	0.742461875729132	2
Butylidenephthalide	188.08373	189.0911405	0.58745454	[M+H]+	6.132	0.9071020925964	2
Caffeate	180.04226	163.039083	0.68328598	[M+H-H2O]+	5.336	0.920715613644197	1
CARBETAPENTANE	333.23039	334.2376699	0.060236469	[M+H]+	5.912	0.886889003469096	2
Compactin diol lactone	306.18311	307.1902175	0.62886783	[M+H]+	6.522	0.925456386297116	2
D(+)-Raffinose	504.16904	503.1620743	0.662998363	[M-H]-	1.525	0.972810696183114	1
D-Fructose	180.06339	225.061583	0.03870919	[M+HCOOH-H]-	1.396	0.749526105965105	1
DGMG 18:3	676.36701	675.3600827	0.551023559	[M-H]-	8.317	0.552415676127497	2
Dibutyl maleate	228.13616	251.1253965	0.159790171	[M+Na]+	6.434	0.949392000440422	1
Dichotosinin	478.1839	477.1769893	0.814071475	[M-H]-	5.967	0.654091164526166	2
Esculetin	178.02661	177.0192726	0.210174063	[M-H]-	5.472	0.978326594193745	1

Fabianine	219.16231	220.1697472	0.625869475	[M+H]+	5.657	0.919276926706467	2
Gastrodin	286.10525	331.1036319	0.635687063	[M+HCOOH-H]-	4.2	0.731768898614077	1
Indolelactic acid	205.07389	188.0707223	0.644912456	[M+H-H2O]+	5.35	0.958652686394631	1
Inositol	180.06339	179.0560626	0.152431274	[M-H]-	1.424	0.963905411911243	1
Isoscopoletin	192.04226	193.0497179	0.822458807	[M+H]+	5.649	0.895230321014951	1
L-Malate	134.02152	133.0140183	1.504704797	[M-H]-	1.563	0.970464181933722	1
Loliolide	196.10995	197.1173937	0.732854988	[M+H]+	5.801	0.969700081324052	2
L-Phenylalanine	165.07898	166.0863615	0.493510416	[M+H]+	5.082	0.99989476370608	1
L-Proline	115.06333	116.070891	2.268001265	[M+H]+	1.512	0.999909305662437	1
Luteolin 7-O-(6"-malonylglucoside)	534.10096	535.1087527	0.922462279	[M+H]+	5.75	0.900436548725947	2
Maleic acid	116.01096	115.0035198	1.208494301	[M-H]-	2.443	0.996605703843294	1
Maryal	418.22028	417.213523	1.29832845	[M-H]-	5.87	0.856659831592542	2
Methyl pentadecanoate	256.24023	255.2330173	0.340713665	[M-H]-	10.428	0.508039263899178	1
MG(0:0/18:3(6Z,9Z,12Z)/0:0)	352.26136	375.2503988	0.457738033	[M+Na]+	9.052	-	3
Moracin N	310.12051	311.1276055	0.659541634	[M+H]+	6.617	0.981886080400093	1
Morin	302.04265	303.0498617	0.292494951	[M+H]+	5.627	0.978713551678107	1
Morusimic acid C	491.30943	492.3170761	0.704368375	[M+H]+	5.595	0.5921343054556	2
Morusimic acid F	329.25661	330.2638712	0.117713452	[M+H]+	5.755	0.743321019899525	2
Mulberrin	422.17294	421.1658152	0.414964607	[M-H]-	7.008	0.968231556708341	1
N-acetylphenylalanine	207.08954	206.0821798	0.290780886	[M-H]-	5.69	0.981239765641245	1
N-decanoylglycine	229.16779	230.1751792	0.389019652	[M+H]+	5.23	0.74324066234048	2
N-Fructosyl tyrosine	343.12672	344.1341485	0.374496883	[M+H]+	2.548	0.590502517523786	2
Nicotiflorin	594.15848	593.1517378	0.938832209	[M-H]-	5.71	0.947057904649298	1
Nicotinamide	122.04801	123.0555213	1.731166538	[M+H]+	2.071	0.995547960836531	1
O-methylmalonylcarnitine	261.12124	262.1285987	0.224673006	[M+H]+	2.019	0.901601624121437	2
O-Succinyhomoserine	219.07429	200.0564094	0.368016776	[M-H-H2O]-	3.073	0.521386642771388	1
Pandangolide 1	244.13108	243.1238283	0.197932492	[M-H]-	5.921	0.92683010751264	2
Perillyl alcohol	152.12011	135.1169928	1.201600183	[M+H-H2O]+	5.847	0.934546631687312	1
Phaeophorbide b	606.24784	607.2553802	0.39615497	[M+H]+	9.487	-	3
Picolinic acid	123.03203	124.0395281	1.610128996	[M+H]+	1.983	0.704493131520852	1
Pseudotropine	141.11536	142.1227628	0.728424858	[M+H]+	1.529	0.817826206520424	2
Pyridoxine	169.07389	170.081332	0.840096995	[M+H]+	2.29	0.860166233519608	1
Pyropheophorbide-a	534.26309	535.2706451	0.477458798	[M+H]+	11.382	0.993257250973331	1
Quercetin 3-O-neohesperidoside	610.15339	609.1466857	0.97625323	[M-H]-	5.603	0.947126194736161	1
Quinic acid	192.06339	191.0560302	0.311214449	[M-H]-	1.492	0.999418529182326	1
Rhodiosin	610.15339	611.1611493	0.752770109	[M+H]+	5.599	0.5971620772075	1
Rosmarinic acid	360.08452	359.0774934	0.75925871	[M-H]-	5.247	0.631992775705337	1
Salicylic acid	138.0317	137.0242492	1.092773957	[M-H]-	6.055	0.999993228755003	1
Soraphen O	308.19876	307.1916375	0.575889196	[M-H]-	6.531	0.911359420742025	2
spinosyn macrolactone	422.26684	423.2739652	0.413871629	[M+H]+	8.413	0.896112231246236	2
Tetracentronside B	520.19447	519.1876138	0.853189227	[M-H]-	5.962	0.642479626685635	2
Traumatic Acid	228.13616	227.1288477	0.053771502	[M-H]-	6.435	0.999994694733819	1

Tricin 7-glucuronide	506.10604	505.0991043	0.719827908	[M-H]-	5.648	0.950805896159047	2
Umbelliferone	162.0317	161.024329	0.438050795	[M-H]-	5.7	0.829017686873946	1
Undecylenic acid	184.14633	183.1389289	0.549281715	[M-H]-	6.434	0.999994019777457	1
