# Supplementary figures and images for: Regional Variation in Mulberry Leaf Metabolites: A Combined Metabolomic and Environmental Analysis of Biosynthetic Drivers
Source: Metabolites. 2025 Nov 6;15(11):728. doi: 10.3390/metabo15110728 (PMC12654259; doi:10.3390/metabo15110728)

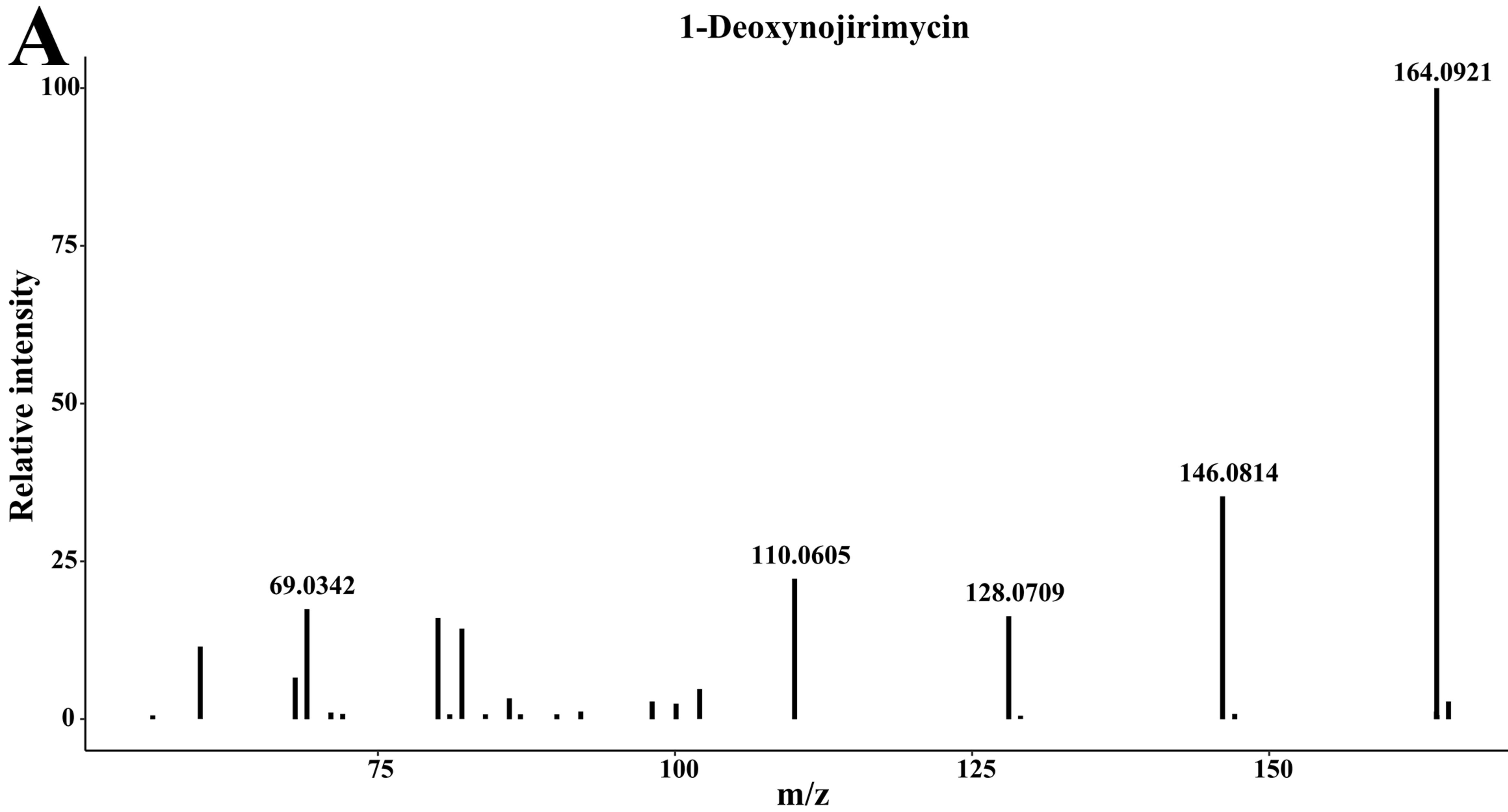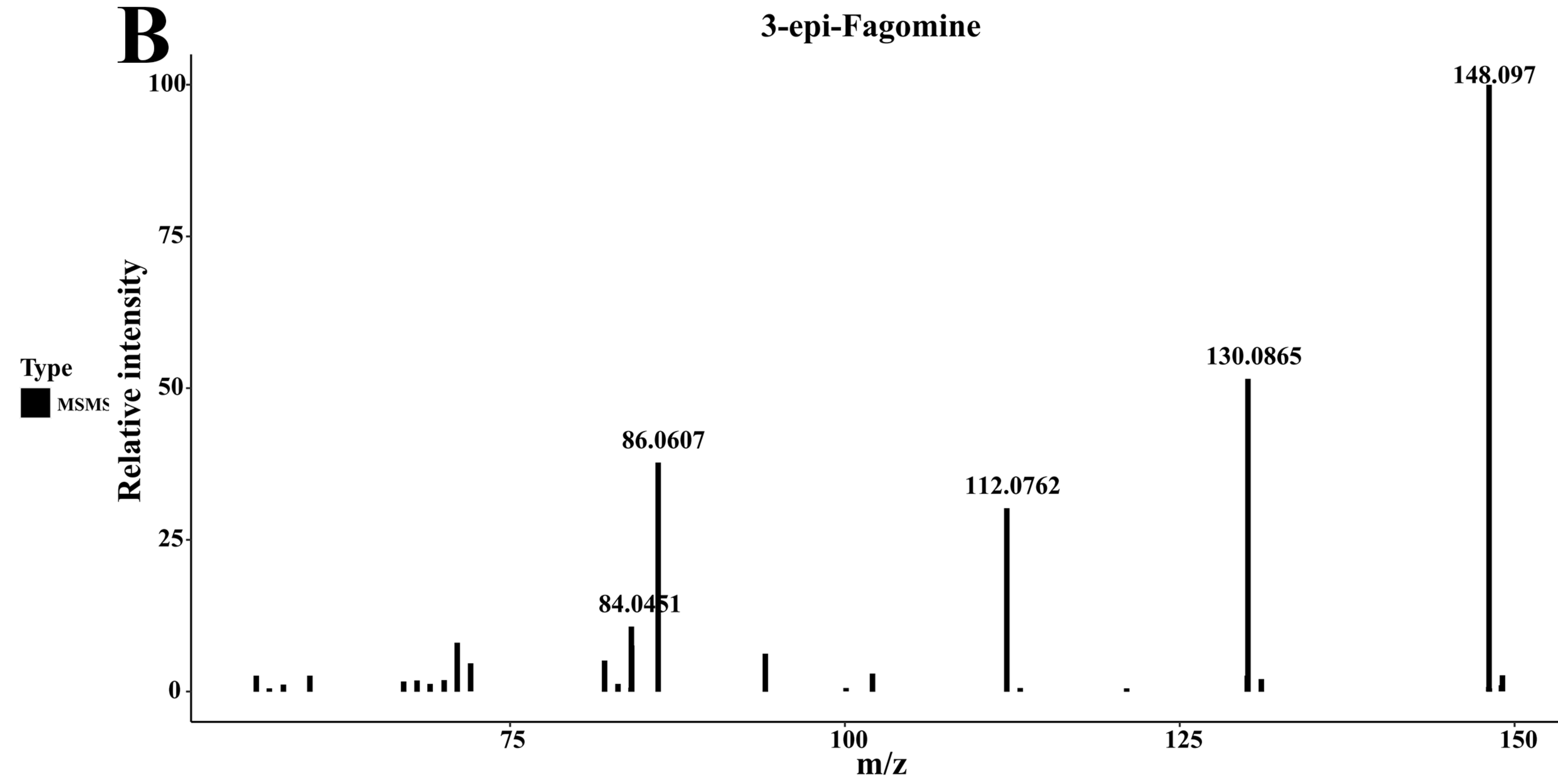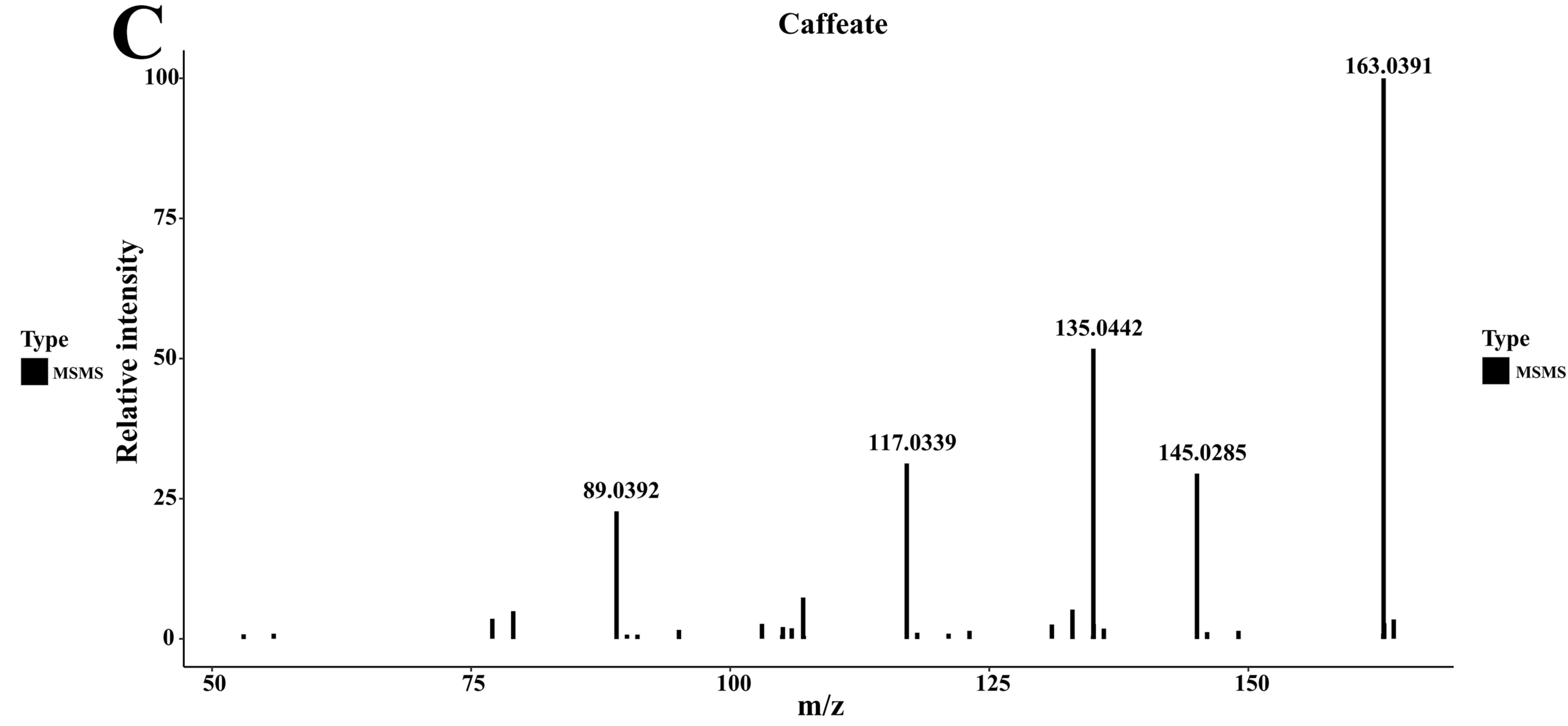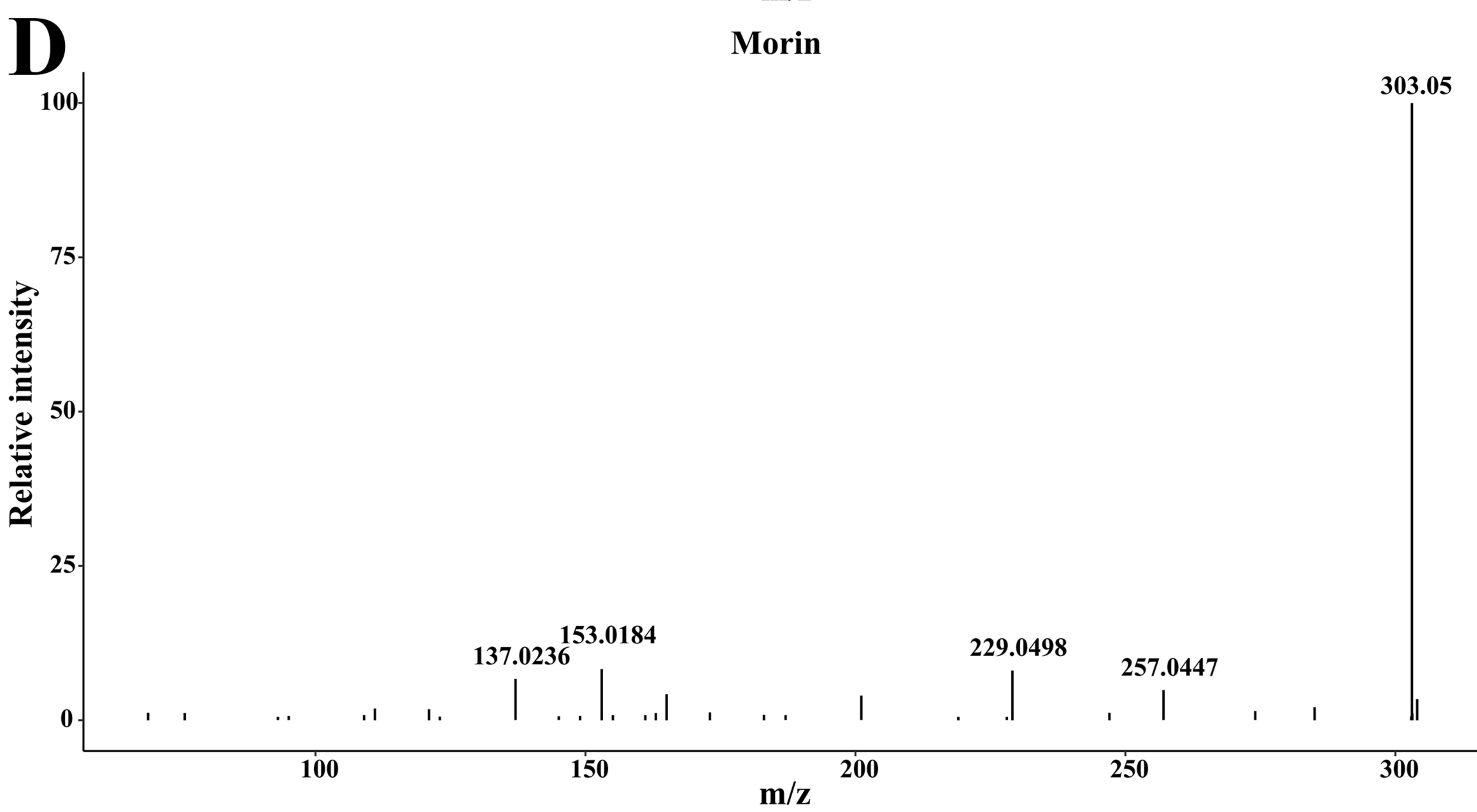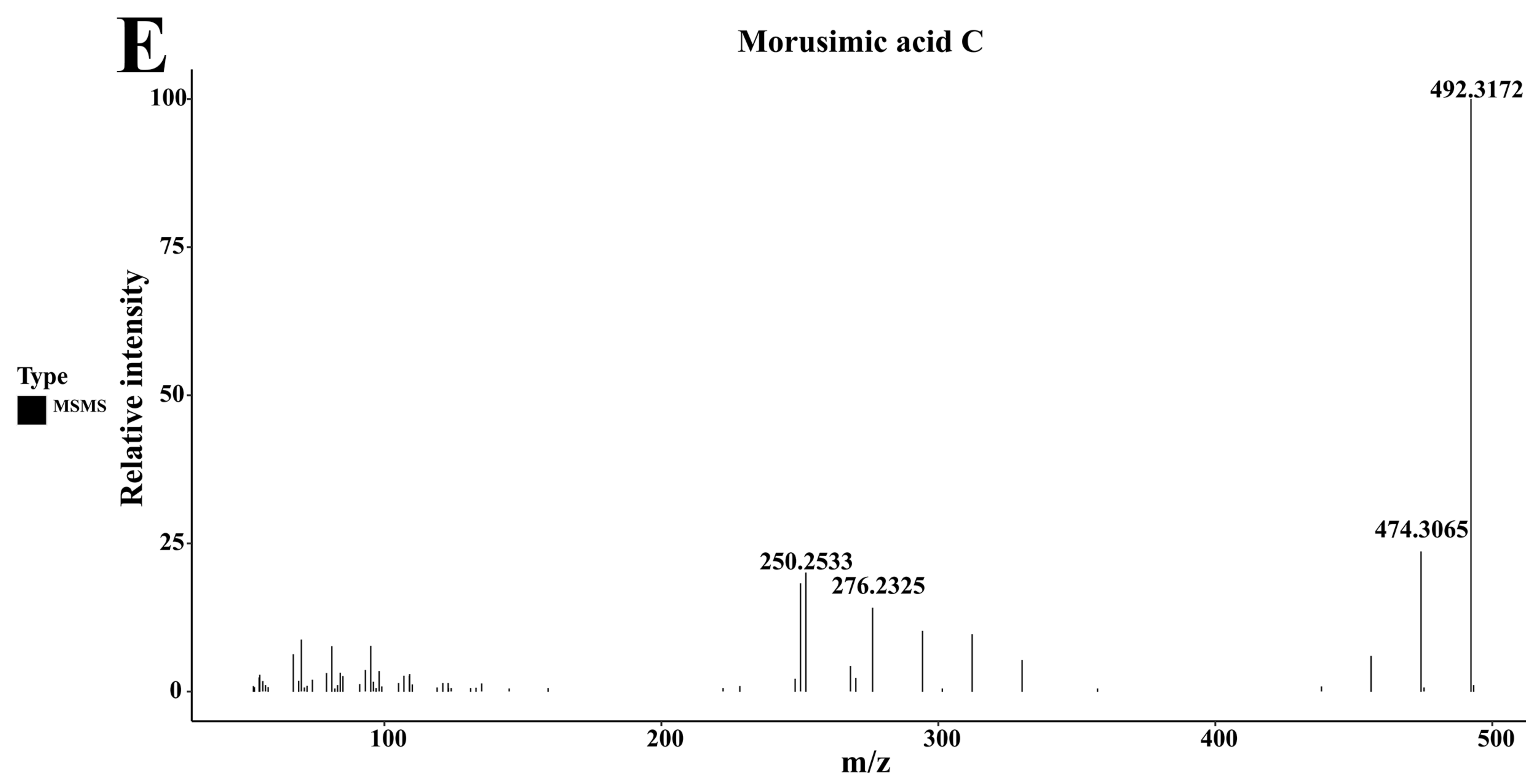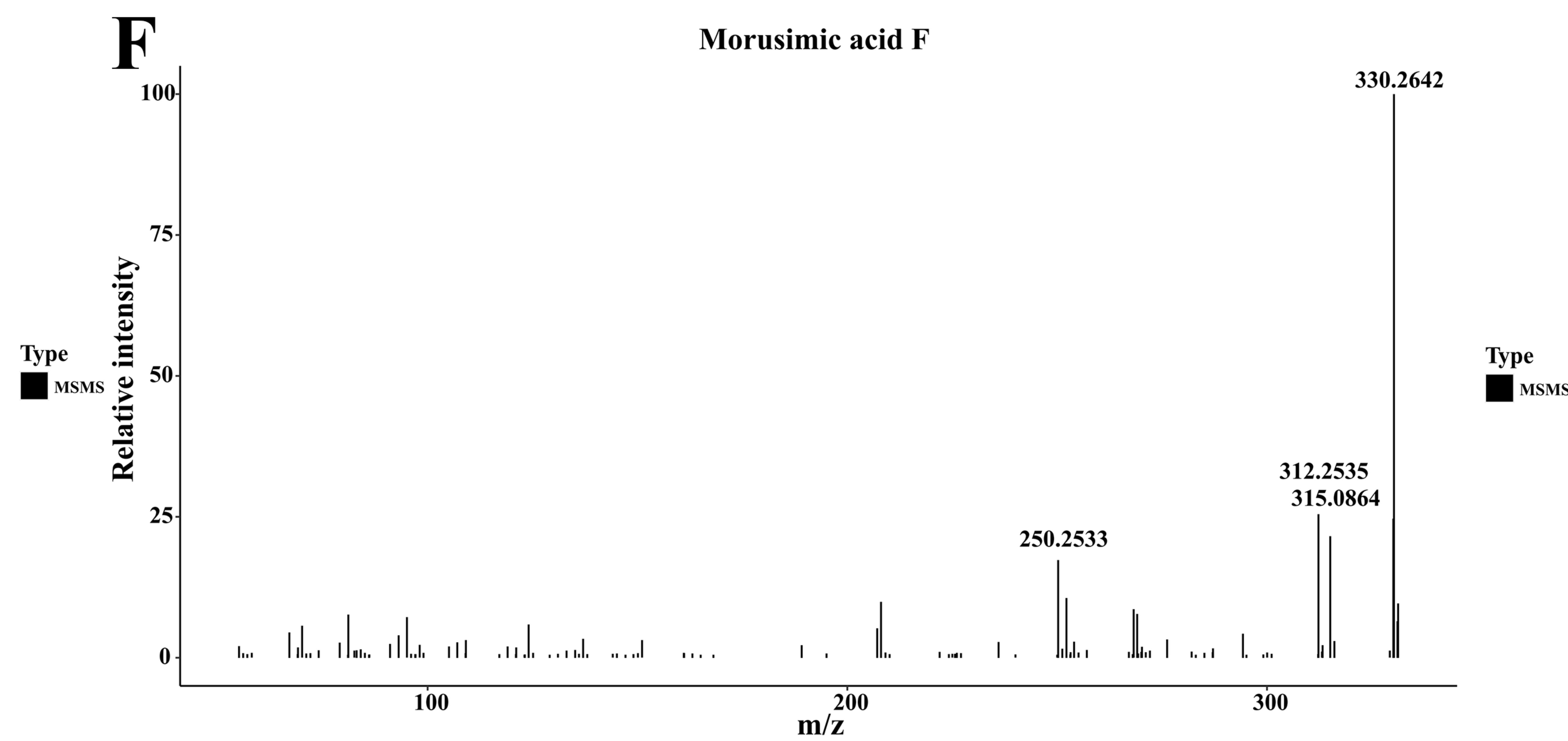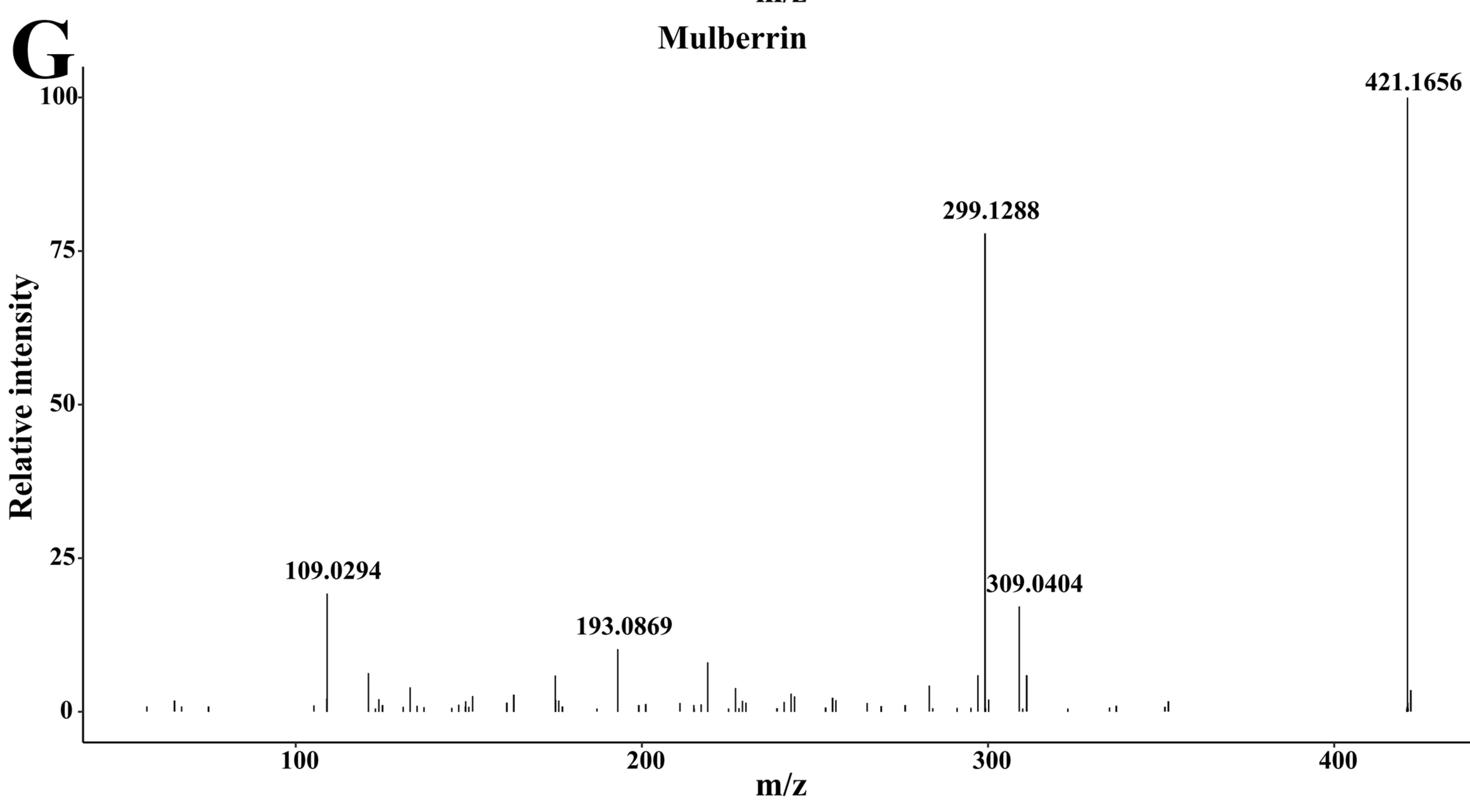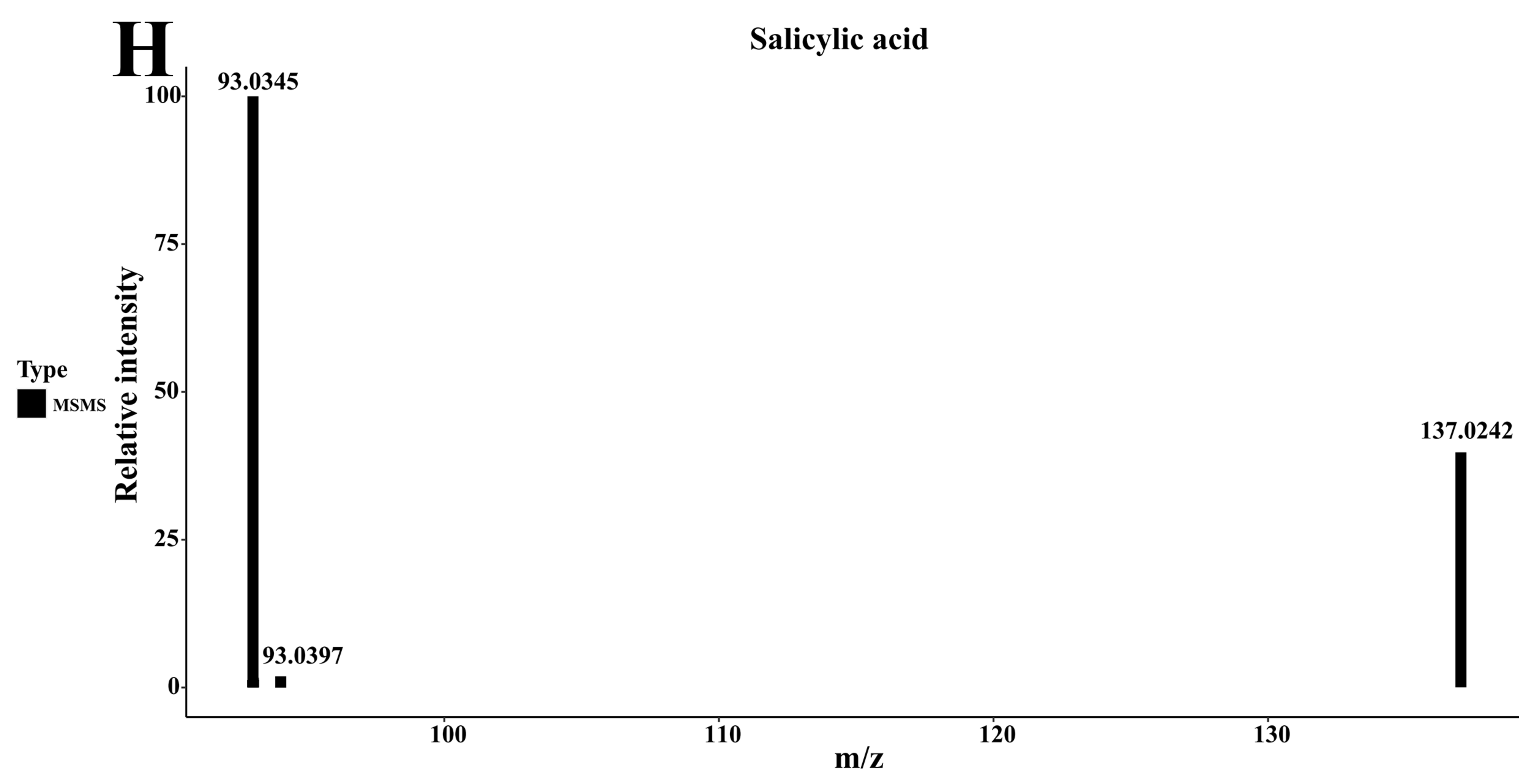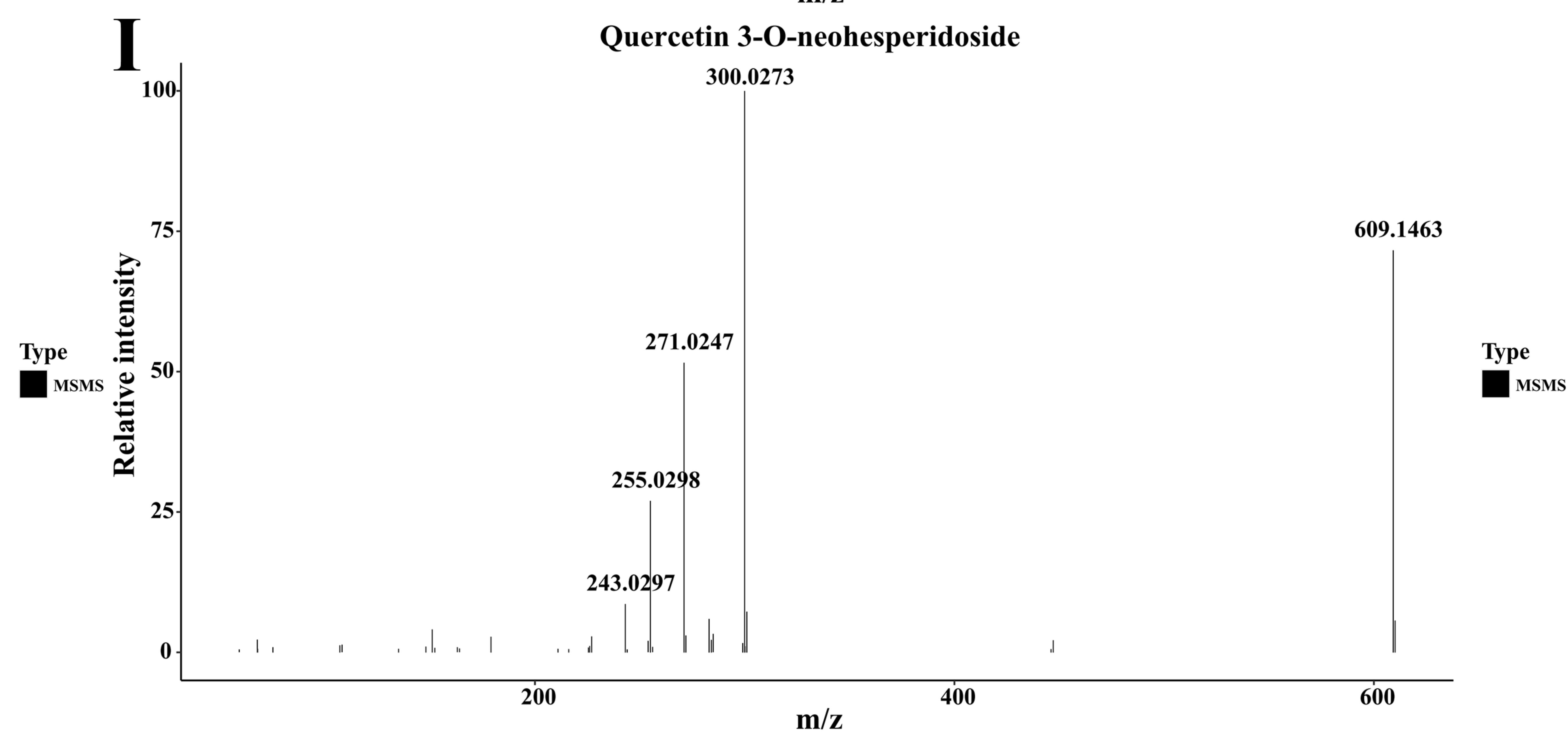

Supplement: Supplementary file 1 [file metabolites-15-00728-s001.zip › Supplementary Figure 3.pdf]
