# Supplementary material for: Regional Variation in Mulberry Leaf Metabolites: A Combined Metabolomic and Environmental Analysis of Biosynthetic Drivers
Source: Metabolites. 2025 Nov 6;15(11):728. doi: 10.3390/metabo15110728 (PMC12654259; doi:10.3390/metabo15110728)
Supplement: Supplementary file 1 [file Supplementary Figure 1.pdf › Supplementary Figure 1.pdf]

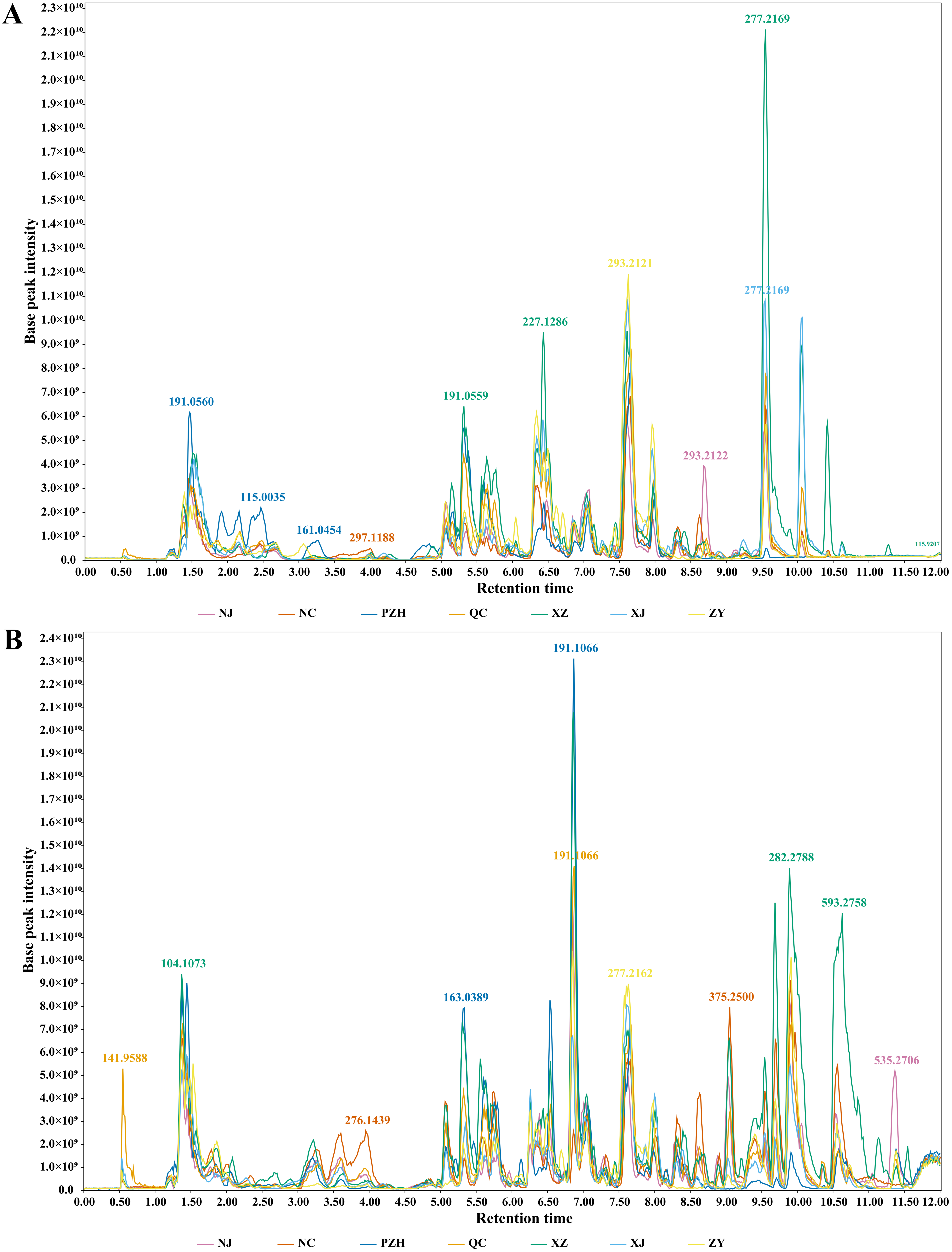
A
B
Base peak intensity
Retention time
191.0560
115.0035
161.0454
297.1188
191.0559
227.1286
293.2121
293.2122
277.2169
277.2169
115.9207
141.9588
104.1073
276.1439
163.0389
191.1066
191.1066
277.2162
375.2500
282.2788
593.2758
535.2706
NJ
NC
PZH
QC
XZ
XJ
ZY
