# Supplementary material for: Regional Variation in Mulberry Leaf Metabolites: A Combined Metabolomic and Environmental Analysis of Biosynthetic Drivers
Source: Metabolites. 2025 Nov 6;15(11):728. doi: 10.3390/metabo15110728 (PMC12654259; doi:10.3390/metabo15110728)
Supplement: Supplementary file 1 [file Supplementary Figure 2.pdf › Supplementary Figure 2.pdf]

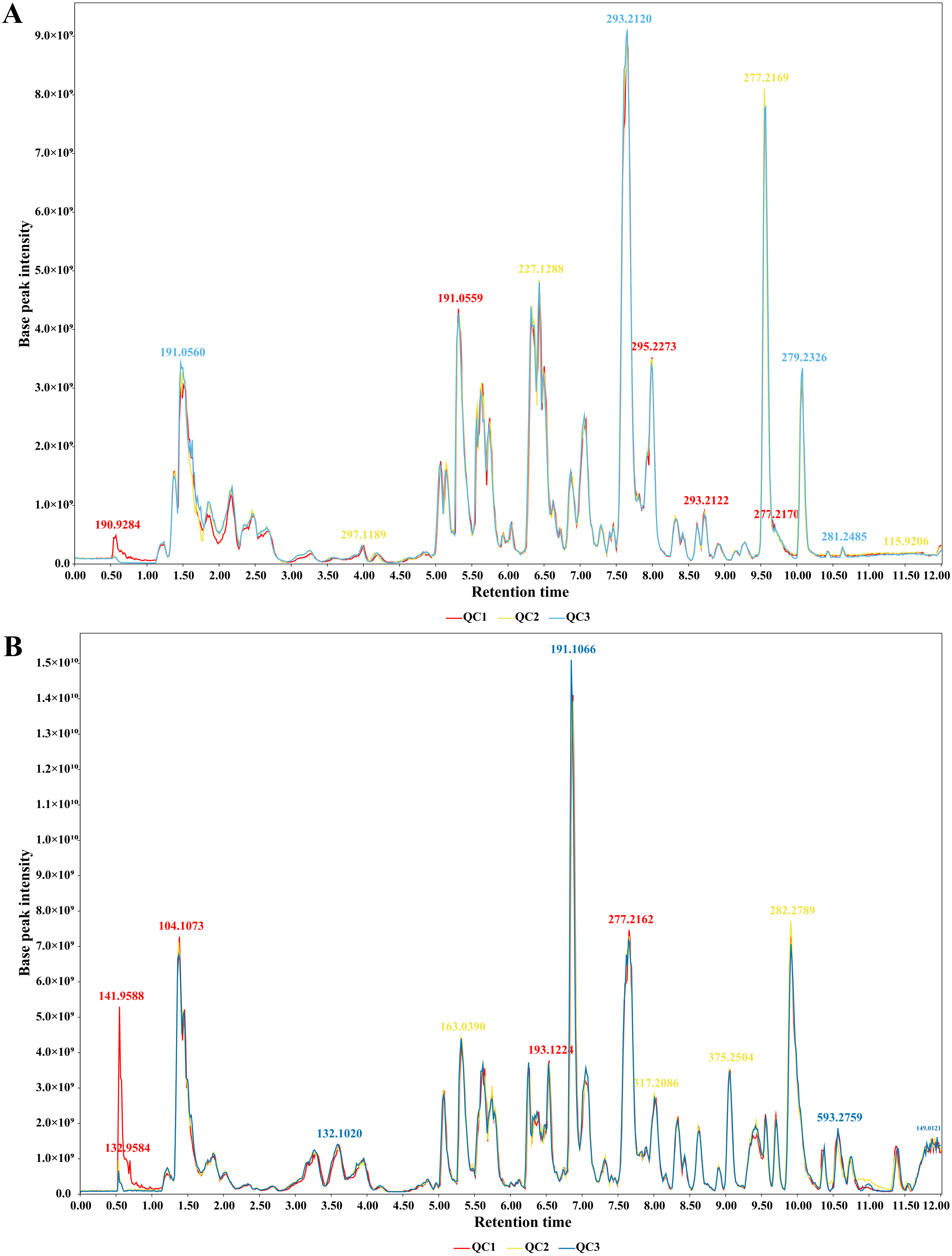

A
293.2120
277.2169
227.1288
191.0559
295.2273
279.2326
191.0560
293.2122
277.2170
190.9284
297.1189
281.2485
115.9206
Base peak intensity
Retention time
QC1
QC2
QC3
B
191.1066
277.2162
282.2789
104.1073
141.9588
163.0390
193.1224
317.2086
375.2504
132.1020
593.2759
132.9584
149.0121
Base peak intensity
Retention time
QC1
QC2
QC3
